# Table-top three-dimensional photoemission orbital tomography with a femtosecond extreme ultraviolet light source

**DOI:** 10.1038/s41467-026-74308-1

**Published:** 2026-06-19

**Authors:** Wiebke Bennecke, Thi Lan Dinh, Jan Philipp Bange, David Schmitt, Marco Merboldt, Lennart Weinhagen, Bent van Wingerden, Fabio Frassetto, Luca Poletto, Marcel Reutzel, Daniel Steil, D. Russell Luke, Stefan Mathias, G. S. Matthijs Jansen

**Affiliations:** 1 Ist Institute of Physics, University of Göttingen, Göttingen, Germany; 2 Institute for Numerical and Applied Mathematics, University of Göttingen, Göttingen, Germany; 3 Institute for Photonics and Nanotechnologies CNR-IFN, Padova, Italy; 4 Fachbereich Physik, Philipps-Universität Marburg, Marburg, Germany; 5 mar.quest—Marburg Center for Quantum Materials and Sustainable Technologies, Marburg, Germany; 6 International Center for Advanced Studies of Energy Conversion (ICASEC), University of Göttingen, Göttingen, Germany

**Keywords:** Electronic properties and materials, Imaging techniques

## Abstract

Following electronic processes in molecules and materials at the level of the quantum mechanical electron wavefunction with ångström-level spatial resolution and with full access to its femtosecond temporal dynamics is at the heart of ultrafast condensed matter physics. A breakthrough invention allowing experimental access to electron wavefunctions was the reconstruction of molecular orbitals from angle-resolved photoelectron spectroscopy data in 2009, termed photoemission orbital tomography (POT). This invention opens a route towards ultrafast three-dimensional (3D) POT, with many new prospects for the study of ultrafast light-matter interaction, femtochemistry, and photo-induced phase transitions. Here, we develop a synergistic experimental-algorithmic approach to realize the first 3D-POT experiment using a short-pulse extreme ultraviolet light source. We combine a new variant of photoelectron spectroscopy, namely ultrafast momentum microscopy, with a table-top spectrally-tunable high-harmonic generation light source and a tailored algorithm for efficient 3D reconstruction from sparse, undersampled data. This combination dramatically speeds up the experimental data acquisition, while at the same time reducing the sampling requirements to achieve complete 3D information. We demonstrate the power of this approach by full 3D imaging of the frontier orbitals of a prototypical organic semiconductor adsorbed on pristine Ag(110).

## Abstract (web summary)

The authors report on a demonstration of 3D photoemission orbital tomography capable of ultrafast imaging of orbitals at atomic scales. This opens towards 3D time-resolved orbital imaging, allowing study of exciton and orbital dynamics.

## Introduction

**Fig. 1:**
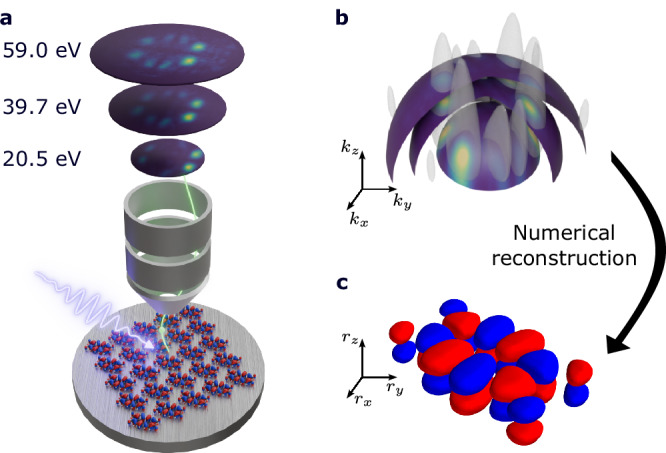
Schematic overview of a three-dimensional photoemission orbital tomography experiment. **a** A photon with energy *hν* photoexcites an electron out of the highest occupied molecular orbital of a well-ordered molecular layer, and a photoemission momentum microscope is used to record the full momentum- and energy-dependent photoelectron spectrum. Repeating this measurement for several *hν* yields a 3D fingerprint of the molecular orbital. **b** According to the plane-wave model of photoemission, each photoelectron's kinetic energy yields the intensity distribution on a hemispherical shell in the 3D momentum space of the molecular orbital represented by the gray rods. **c** Isosurface representation of the numerical reconstruction of the three-dimensional HOMO of PTCDA from the measured sparse data recorded at only seven photon energies. Red and blue denote regions of opposite sign.

The quantum mechanical electron wavefunction determines fundamental properties of matter such as electronic structure, chemical bonding, and light-matter interaction. In consequence, an in-depth characterization of its properties offers unique insight into electronic and optoelectronic functionalities of materials. Photoemission orbital tomography (POT) has appeared as a uniquely sensitive probe for the electronic wavefunction in organic molecular systems^1^. Nowadays, POT allows studying optically excited^2–4^ and excitonic states^5–10^, characterizing molecular structure, absorption geometry, and interlayer hybridization in hybrid interfaces^11–13^ and the direct imaging of molecular orbitals independent of theoretical calculations^1,14–16^. Among a range of techniques that provide access to the real-space distribution of the electron orbitals^17,18^, POT and the related gas-phase molecular orbital tomography^19,20^ stand out furthermore due to a unique capability: they provide non-invasive access to the intrinsic three-dimensional shape of the molecular orbital^21,22^. The mechanism by which three-dimensional information is acquired is the same for both methods: full three-dimensional electron momentum distributions are acquired by recording photoelectron spectra for a range of photon energies. As sketched in Fig. 1, analysis of these data enables imaging the full three-dimensional (3D) molecular orbital in real space with sub-ångström resolution.

However, while all of these studies have yielded ground-breaking results, a long-awaited extension of POT would combine full 3D orbital reconstruction with a tunable femtosecond extreme ultraviolet (EUV) light source that would not only enable three-dimensional orbital imaging, but also time-resolved studies at the laboratory scale. Such an experiment has many potential applications in different research areas of (ultrafast) condensed matter physics, for example, for the study of orbital hybridization in organic-inorganic heterostructures^13,23^, for time-resolved studies of exciton dynamics in organic semiconductors^5,6^, and for motion-induced orbital dynamics^18,24^.

For this reason, the first 3D photoemission orbital tomography (3D-POT) studies have been undertaken^12^ and successfully imaged the two highest occupied molecular orbitals (HOMOs) of PTCDA (full name: perylene-3,4,9,10-tetracarboxylic dianhydride) adsorbed on the Ag(110) surface^21,22^. However, the nature of 3D-POT measurements, requiring photon-energy-dependent data collection in the EUV range, has so far prevented widespread adoption and restricted the experiments to large-scale synchrotron facilities. Moreover, in order to measure complete and high-resolution momentum distributions for a given orbital, 3D-POT seemingly relies on recording two-dimensional photoemission momentum maps for a large number of photon energies^21,22^. While this tedious data acquisition was already sped up through the use of momentum microscopy^16,22^, the vast amount of data needed to reconstruct 3D information and, for time-resolved experiments, the need to scan several pump-probe delays has so far strongly limited the potential of 3D-POT.

In this article, we overcome all of these limitations and demonstrate how 3D-POT can be performed in the laboratory at dramatically reduced measurement durations. To achieve this, we have combined time-of-flight (ToF) photoemission momentum microscopy^25^ with a photon-energy-tunable femtosecond high-harmonic generation (HHG) light source covering the photon energy range from 13 to 71 eV^26^. This ToF-based setup, which does not rely on scanning the energy or momentum of the photoelectrons, allows us to collect the photon-energy-dependent momentum distributions needed for 3D reconstruction much more efficiently. Importantly, we complement this experimental strategy with an advanced numerical reconstruction algorithm that requires much less photon energy for full 3D orbital imaging^27^. All in all, this joint experimental-algorithmic development establishes a lab-scale streamlined 3D-POT, which we benchmark by 3D imaging of the frontier molecular orbitals of PTCDA/Ag(110). Our results illustrate the potential of table-top 3D-POT for systematic studies of orbital hybridization and suggest a viable route towards future femtosecond time-resolved orbital tomography experiments.

## Results and discussion

### Light source for an ultrafast 3D-POT experiment

The requirements for the light source in a 3D-POT experiment are directly determined from the desired resolution of the reconstructed molecular orbital in 3D-POT. According to the commonly used plane-wave approximation of the photoemission process^1,28^, the measured photoelectron current *I*_*hν*_ in a POT experiment can be expressed as

1 $${I}_{h\nu }({{\bf{k}}})=| {{\bf{A}}}\cdot {{\bf{k}}}{| }^{2}\,| {{\mathcal{F}}}(\psi )({{\bf{k}}}){| }^{2}\,\delta ({E}_{{{\rm{I}}}}+{E}_{{{\rm{kin}}}}-h\nu )\,.$$

This equation highlights the well-known relation that the momentum-resolved photoelectron distribution is proportional to the Fourier transform ${{\mathcal{F}}}$ of the real-space molecular orbital *ψ*, modulated by a weakly varying polarization factor ∣**A**⋅**k**∣. Here, **A** is the vector potential of the incident (ionizing) light, which has photon energy *hν*, and **k** is the photoelectron momentum. The Dirac delta in Eq. (1) expresses the energy conservation in terms of the photon energy *hν*, the orbital ionization energy *E*_I_, and the photoelectron kinetic energy *E*_kin_. As the latter is proportional to *ℏ*^2^*k*^2^, it follows that an angle-resolved photoelectron spectroscopy (ARPES) measurement at a fixed photon energy measures the value of $| {{\mathcal{F}}}(\psi )({{\bf{k}}})|$ on a hemisphere in momentum space (see Fig. 1b). In the absence of an inner potential, the radius of this hemisphere can be approximated by

$$k({{{\AA }}}^{-1})| \approx 0.514\sqrt{{E}_{{{\rm{kin}}}}({{\rm{eV}}})}.$$

In POT, this relation implies that photon energies of at least 15–20 eV are necessary to probe the features of valence orbitals in *π*-conjugated organic molecules, as their brightest photoemission signatures usually appear around ≈2 Å^−1^. Similarly, the resolution in 3D (and 2D) orbital imaging can be directly estimated from this relation, and it follows that the photon energy must be approximately quadrupled to achieve a doubling of the final image resolution. It can thus be concluded that ångström-scale 3D-POT requires a broad range of EUV photon energies, starting at 15 eV and extending upwards of 60 eV. These photon energies can be generated efficiently using laser-based HHG^29,30^, which conveniently provides a short-pulse table-top solution that enables time-resolved spectroscopies, too^2,25,31–35^.

To generate HHG over the desired spectral window, we tightly focus 65 W output of our Yb-fiber laser amplifier (35 fs pulses, 500 kHz repetition rate, 130 *μ*J pulse energy) using an *f* = 7.5 cm lens into an argon gas jet, which has sufficiently high ionization potential to achieve a >71 eV cutoff while also affording good HHG efficiency. In order to isolate individual high harmonics and to apply these in the photoelectron momentum microscope, we implemented a grating-based EUV monochromator in grazing-incidence, off-plane diffraction geometry^26,32,36^ (see Supplementary Information). In this way, a high photon efficiency can be achieved for both *s* and *p* polarization, while the grating line spacing can be minimized efficiently to reduce the effects of geometric temporal broadening of the femtosecond EUV pulses^37^. The design of the single-grating monochromator used in this study is shown in Supplementary Fig. 1 and described in detail in the “Methods” section “ Ultrafast EUV monochromator”. In short, a selection of four different plane gratings (100, 300, 400, 600 lines/mm, respectively) allows us to choose any harmonic from our HHG light source between 13 eV and 71 eV with minimized temporal broadening and high transmission efficiency (see Supplementary Figs. 2 and 3). Thus, we can perform time-resolved momentum microscopy using 25 different photon energies with a spacing of 2.4 eV within this energy range.

### Table-top 3D-POT data collection

We demonstrate the 3D-POT capabilities for the prototypical hybrid interface of PTCDA adsorbed on the Ag(110) surface^14,15,21,22^. For this interface, the first PTCDA monolayer adsorbs in a highly ordered brick-wall structure, where furthermore the lowest unoccupied molecular orbital (LUMO) of the gas-phase molecule is occupied due to charge transfer from the substrate^38^. Thus, the ToF momentum microscope allows to measure orbital fingerprints of both the HOMO and the LUMO simultaneously^16^.

In order to have a comprehensive data set to test our setup and the developed 3D-POT algorithms, we measured the full momentum-energy-resolved photoelectron spectrum of PTCDA/Ag(110) for ten different EUV photon energies ranging from 20.5 eV to 63.8 eV (cf. Methods, Table 1). Each spectrum was recorded within 2 h at a typical count rate of 2 × 10^5^ photoelectrons per second (integrated over the full spectrum, laser repetition rate of 500 kHz). We reiterate that the ToF-based momentum microscope records the full 2D momentum and kinetic energy of each individual electron that is accepted into the microscope. In practice, a single data set contains 3D (*k*_*x*_, *k*_*y*_, *E*_kin_) data in an energy range of about 5 eV and over a circular momentum region with a radius of about 3 Å^−1^, so that no additional scanning in kinetic energy or momentum is necessary. In addition, we emphasize that we measured ten photon-energy-dependent data sets to be able to thoroughly test our algorithms, but that one of the most important and surprising results of our work is that, by using our iterative reconstruction algorithm, even just four data sets can be sufficient for a full 3D reconstruction of the orbitals.

**Table 1:** Overview of the parameters of measured photon energies: Used grating, energy, and momentum resolution (FWHM) of the respective photoemission data set, calibration, photon energies used for the reconstruction based on 4, 7, and 10 photon energies

Photon energy (eV)	Grating (lines/mm)	Energy resolution (meV)	Momentum resolution (Å^−1^)	Calibration factor	4 *hν*	7 *hν*	10 *hν*
20.5	300	81 (3)	0.073 (1)	0.19 (2)	x	x	x
25.3	300	58 (2)	0.073 (7)	0.137 (5)		x	x
30.1	300	88 (2)	0.062 (3)	0.189 (5)	x	x	x
34.9	300	106 (2)	0.077 (9)	0.213 (9)		x	x
39.7	300	129 (3)	0.102 (9)	0.22 (2)			x
44.5	300	132 (2)	0.07 (2)	0.22 (4)		x	x
49.4	400	240 (5)	0.13 (2)	0.30 (1)	x		x
54.2	400	310 (20)	−	0.52 (7)		x	x
59.0	600	202 (3)	−	0.33 (2)			x
63.8	600	183 (3)	−	0.23(8)	x	x	x

**Fig. 2:**
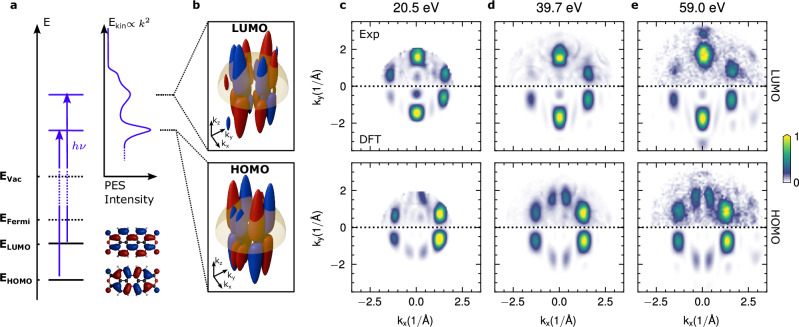
Exemplary 3D-POT data of the frontier orbitals of PTCDA adsorbed on Ag(110). **a** Energy alignment of the vacuum and the Fermi level and the HOMO and LUMO states, indicating that the LUMO shifts below the Fermi level and is occupied due to charge transfer from the silver surface. Electrons from these states are photo-excited using EUV light with photon energy *hν*, giving rise to a photoelectron spectrum such as sketched in the top right. The bottom right of **a** shows isosurfaces of the HOMO (bottom) and LUMO (top) wavefunctions of PTCDA calculated by DFT^39^. **b** For each photon energy, we record the intensity on a hemisphere (yellow shaded half-sphere) in 3D momentum space, such that the radius of the hemisphere ∣*k*∣ is given by $\sqrt{h\nu -{E}_{{{\rm{HOMO}}}/{{\rm{LUMO}}}}}$. **c**–**e** Typical photoemission momentum maps recorded at photon energies *hν* = 20.5 (**c**), 39.7 (**d**), and 59.0 eV (**e**) for the HOMO (bottom row) and LUMO (top row). In each momentum map, the top half (*k*_*y*_ > 0) shows the experimental data, while the bottom half (*k*_*y*_ < 0) shows the predicted momentum fingerprint for a gas-phase PTCDA molecule from density functional theory retrieved from ref. ^39^. Each momentum fingerprint has been normalized to the maximal intensity feature for visualization only.

Before we can start the real-space reconstruction, we first need to determine the energy of each orbital in the photoelectron spectrum (Fig. 2a) and extract the corresponding 2D momentum fingerprint for each used photon energy, which is then mapped to hemispherical shells as indicated in Fig. 2b. To do so, we model the photoelectron spectrum at each momentum (*k*_*x*_, *k*_*y*_) by a Gaussian lineshape of the molecular orbital fingerprint overlapping with a smooth Fermi–Dirac distribution, and fit the relative amplitudes for each position. A full description of the fit routine to extract the orbital momentum fingerprint, i.e., to remove all background other than the intensity of the orbital itself, is given in the “Methods” section “Background subtraction” and Supplementary Information (see Supplementary Fig. 4 for an exemplary data set). Fig. 2c–e shows the background-corrected momentum maps of the LUMO and HOMO for three exemplary photon energies. The experimental data (upper half of each panel) is compared to simulated momentum maps of the gas-phase PTCDA molecule based on Kohn–Sham (KS) orbitals retrieved from ref. ^39^ (lower half of each panel), and is in excellent agreement. The observed left-right asymmetry in both experiment and theory stems from the polarization factor ∣**A**⋅**k**∣^2^ in Eq. (1), with the light impinging from the left. To retrieve the momentum space amplitude $| {{\mathcal{F}}}(\psi )({{\bf{k}}})|$ required for reconstruction, the data are first symmetrized and subsequently divided by the symmetrized polarization factor. After normalization of the extracted momentum maps to the measured photon flux, the data are ready for iterative reconstruction of the real-space molecular orbitals.

### 3D reconstruction of the orbitals

In order to carry out efficient and time-resolved 3D-POT, the development of a tailored reconstruction algorithm for the 3D orbital from the hemispherical shell photoelectron data is indispensable. Indeed, the most generally applicable method that has been demonstrated to date^22^ relies on dense photon energy sampling to acquire POT data on hemispherical shells that are spaced closely enough that linear interpolation may be used. However, as argued in ref. ^27^, interpolation of the intensity-only POT data ($\propto | {{\mathcal{F}}}(\psi )|$) is susceptible to systematic errors around zero crossings in the 3D momentum space distribution ${{\mathcal{F}}}(\psi )$. Such errors can be completely avoided if the reconstruction of ${{\mathcal{F}}}(\psi )$ is incorporated in an algorithm that recovers both amplitude and phase (i.e., sign) simultaneously. It is also straightforward to expect that such an algorithm for 3D orbital reconstruction might require less data overall to achieve similar results, which has a direct impact on the experimental design and measurement duration. Indeed, we have recently reported such a minimalist-approach algorithm, which was tested on simulated data^27^, and we have here further developed the algorithm to be applied to our first experimental 3D-POT momentum microscopy data set.

**Fig. 3:**
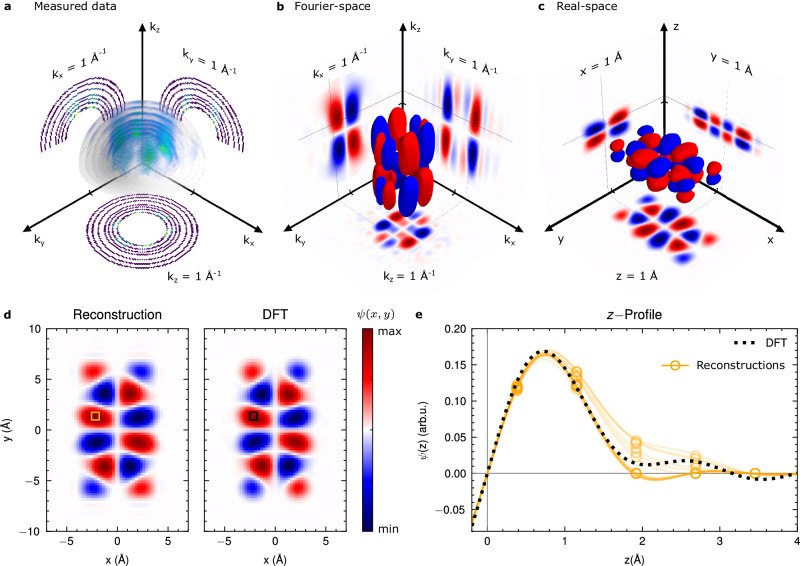
3D orbital imaging of the PTCDA HOMO using only seven photon energies. **a** Here, the raw measurement data is visualized by a cloud of semi-transparent voxels lying on hemispherical shells (see “Methods”, Table 1 for the exact photon energies). The sparsity of the data is emphasized by slices through the data at *k*_*x*/*y*/*z*_ = 1 Å^−1^, showing that only a fraction of the voxels in momentum space contain measurement data, whereas most voxels have no data (indicated by white or transparent in the slices and 3D plot, respectively). **b**, **c** The reconstructed HOMO in momentum space and real space, respectively. Red and blue denote regions of opposite sign. Evidently, the reconstruction algorithm fully recovers the momentum-space amplitudes and phases, and gives a direct view of the full 3D molecular orbital. **d** Comparison of the in-plane (*x*, *y*) structure of the reconstructed orbital at *z* = 0.5 Å with the prediction from density functional theory (DFT) of the gas-phase molecule^39^. For an equal comparison, a low-pass filter was applied to the orbital matching to the highest accessible momentum in the experiment. **e** Comparison of the reconstructed *z*-dependence of the HOMO (yellow circles; the yellow line shows a Fourier interpolation) with DFT (black dashes), extracted at (*x*, *y*) = (−2, 1.6) Å (see **d**). Since each random initialization of the reconstruction algorithm yields a slightly different orbital, we plot here the *z*-dependence of 17 independent reconstructions with transparency set to 20% (more saturated yellow thus corresponds to multiple reconstructions yielding the same value). Overall, we observe an excellent agreement between experiment and DFT in both the in-plane structure and the *z*-dependence, with particularly good agreement concerning the position of the maximum at *z* = 0.8 Å.

To recover the full 3D momentum space distribution, including phases and amplitudes for those voxels that were not measured directly (cf. Fig. 3), we use the cyclic projections (CP) algorithm that was presented in ref. ^27^. In addition to the experimental data, the algorithm incorporates several constraints on the reconstructed orbital. Namely, we employed the known symmetry in the *x*, *y*, and *z* directions, a loose support of 12 × 18 × 6 Å^3^ (i.e., roughly matching the van der Waals size of the molecule), and voxel sparsity of the molecular orbital (800 voxels, i.e., 26% of the support). The momentum cutoff was set to 4 Å^−1^, corresponding to the photoemission horizon radius for the data measured with the maximum photon energy of 63.8 eV. A more detailed description of the implemented algorithm can be found in the “Methods” section, “3D orbital reconstruction”. In Fig. 3, we show exemplary reconstruction results of the full 3D molecular orbital for a data set comprising seven photon energies (see Table 1 for details). Figure 3a shows the original sparse 3D-POT data for only seven measured hemispheres, and Fig. 3b, c the reconstructed momentum space and real space distributions. We note that we observe a similarly good reconstruction of the LUMO (see Supplementary Fig. 6).

Having imaged the HOMO with full 3D resolution, we take advantage of the fact that the static non-excited HOMO/LUMO orbitals of PTCDA/Ag(110) can be well approximated using density functional theory (DFT) calculations of the isolated (gas-phase) molecule^14,22^, and directly compare our reconstructed orbitals with these theoretical predictions (an identical analysis of the LUMO reconstruction is presented in the Supplementary Information, Supplementary Fig. 6). In Fig. 3d,e, we present a comparison of the reconstructed HOMO orbital with the KS orbital of the gas-phase molecule calculated by DFT^39^. (Note that a more accurate comparison employs Dyson orbitals, but these have been shown to closely match KS orbitals for the present PTCDA system^40,41^.) It is important to note that the reconstructed orbitals from 3D-POT are intrinsically limited due to the finite momentum range that is accessible in the experiment. At a maximum photoelectron kinetic energy of 63.8 eV, the intrinsic spatial resolution is approximately 0.75 Å. For an equal comparison, we therefore apply a low-pass filter matching to this kinetic energy to the KS orbitals.

As can be seen in Fig. 3d, e, this leads to an excellent agreement for both the in-plane (*x*, *y*) and the out-of-plane (*z*) distribution. Additionally, Supplementary Fig. 9 provides a quantitative comparison of measured and reconstructed *k*_*z*_ profiles with DFT calculations, demonstrating excellent agreement across all photon energies. In this context, we note that a slight bending of the adsorbed PTCDA molecule has been reported, with the oxygen atoms positioned ≈0.3 Å closer to the substrate than the carbon backbone^42^. However, this distortion falls below the current spatial resolution of 0.75 Å, indicating that distortion-induced changes to the photoemission orbital tomography data will be very hard to observe. Since we detect no significant photon-energy dependence in the main lobe positions, and reconstructions without enforced *z*-symmetry constraints are consistent with a planar molecular geometry, we conclude that any influence of this bending on the HOMO and LUMO lies below our detection limit. Thus, we present the first realization of a table-top 3D-POT experiment, which, due to the use of ultrashort pulses from HHG, may enable future studies of dynamical processes with femtosecond time resolution.

### Accuracy, reliability, and experimental design for time-resolved 3D-POT

Handling of the complex and vast amount of experimental momentum microscopy data is made possible by the application of a powerful algorithm for 3D orbital image reconstruction that not only recovers the unknown phase but also accurately estimates the not-measured amplitudes in momentum space. However, it is known that not every random initialization of the CP algorithm leads to the same local minimum in the algorithm’s feasibility landscape (i.e., the estimate of the molecular orbital)^27^. In fact, it is likely that many local minima exist, and the image constraints and algorithm settings must be considered carefully to ensure an optimal estimation of the molecular orbital.

**Fig. 4:**
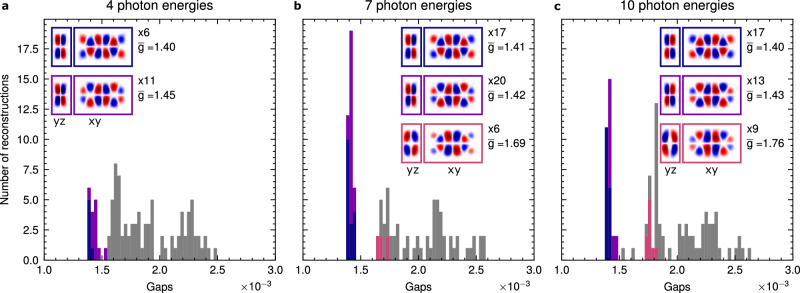
Comparison of the orbital reconstruction results of the HOMO for 4, 7, and 10 photon energies. For each data set, the gap of 100 independent reconstructions was analyzed, and DBSCAN clustering (see “Methods” section) was used to determine the most prominent typical recovered orbitals. From top to bottom, the insets show vertical (yz) and horizontal (xy) slices through the averaged orbital (i.e., the cluster center) of the retrieved clusters, the cluster size, and (10^3^×) the average gap (${\bar{g}}$). The colored boxes correspond to the applied support constraint. The gaps of the corresponding reconstructions are colored accordingly in the histogram. Reconstructions that could not be assigned to a cluster are colored gray. **a** When only four photon energies are used for the reconstruction, the sparse data leads to an increased number of closely-spaced local minima in the feasibility landscape of the algorithm. Notably, the different reconstructions mostly differ by their z-dependency with varyingly pronounced side peaks. **b** When seven photon energies are used instead, the global minimum (optimal solution) becomes more defined. Nevertheless, two solutions (blue and purple insets) remain that are only different by a phase flip in momentum space. As discussed in the main text, this is connected to residual background intensity in the momentum maps. **c** When ten photon energies are used, the stronger measurement constraint leads to an increased gap for faulty reconstructions. The most accurate orbital estimations can therefore easily be selected based on their small gap value.

To address this, we run multiple independent reconstructions and analyze their results in terms of the reconstruction gap (a metric indicating the remaining discrepancy between the various reconstruction constraints). Additionally, we use a clustering algorithm (DBSCAN, see “Methods” section) to extract typical reconstructions from the large, multidimensional output data. The results of this analysis are visualized in Fig. 4a for 3D orbital imaging with data from 4, 7, and 10 photon energies. An identical analysis of the LUMO can be found in Supplementary Fig. 7. We find that the gap, which indicates the remaining discrepancy between the various reconstruction constraints, strongly correlates with the quality of the reconstruction. This is in agreement with ref. ^27^, where the smallest gap was shown to be a good measure for the most likely reconstruction. For the experimental data, however, we find that the minimum gap is not a guaranteed indicator: as shown in Fig. 4, we find two characteristic reconstructions with similarly small gap that differ in the position and shape of the outer lobes (e.g., the blue and purple insets in Fig. 4). In momentum space, this mainly corresponds to a phase flip of the weaker amplitude peaks. A detailed comparison of these reconstructions is given in the Supplementary Information (Section 5 and Supplementary Fig. 8). From this analysis, we find that the lower-gap reconstructions match better with the measured data, and we therefore identified these reconstructions as the most likely true orbital. Incidentally, this choice is also in line with gas-phase DFT calculations. Thus, we emphasize that by analyzing the gap and the reconstructed momentum distribution, accurate 3D orbital images can be extracted from the data with only limited prior information.

The number of local minima in the algorithm’s feasibility landscape and the spread in their gap values can be directly influenced by the various constraints that are applied in the algorithm. A straightforward approach is to add experimental data: Going from 4 to 7 or 10 photon energies, this leads to a more well-defined global minimum, thereby reducing the spread in close-to-optimal reconstructions. Moreover, the additional data further penalize incorrect reconstructions, leading to a larger gap for such reconstructions. This is particularly visible for ten photon energies (Fig. 4c), where proportionally more correct reconstructions are found. With more data, it is therefore possible to discriminate between good and bad reconstructions based on the gap metric. This analysis therefore shows that there is a trade-off between reconstruction reliability and experimental effort: more experimental data increases the reliability. For the present static measurements of the PTCDA/Ag(110) HOMO and LUMO orbitals, we find that measuring at seven photon energies sets a good balance between short experiment time and reconstruction reliability.

On the other hand, the feasibility landscape can also be modified by adapting the support, sparsity, and symmetry constraints. For example, reduction of the support to a more restrictive shape (e.g., 10.5 × 16.5 × 4.5 Å^3^, cf. Supplementary Fig. 5) strongly reduces the spread in reconstructions for the four photon-energy data set and leads to results which are similar to those for seven photon energies with the van der Waals support. This also holds for the symmetry constraint, where we have observed that releasing *z*-symmetry leads to a large number of spurious local minima that so far have prevented a definitive analysis of the potential small bending of the PTCDA molecule^42^. Finally, we note that the choice of optimization algorithm (here CP) will affect the retrieved set of local minima, and more advanced algorithms based on the relaxed Douglas-Rachford algorithm may improve convergence to the global minimum^43–45^.

### Outlook

The above discussion is most critical and important for the experimental design of a time-resolved 3D-POT experiment, which clearly is possible using our short-pulse HHG-based setup. At the femtosecond timescale, where the prediction of excited non-equilibrium orbital wavefunctions is particularly challenging^10,46,47^, the objective is commonly to follow dynamical changes in the electronic structure and orbitals with just a sufficient amount of data per time-step in order to keep the overall measurement time in a feasible range. In our particular case, we can directly see that after a thorough measurement of the static orbitals with about seven measured momentum hemispheres, this knowledge provides a valuable starting point for the dynamic, i.e., time-resolved, measurements. In other words, while a measurement at seven photon energies is necessary to determine the static orbital and its support, the knowledge extracted from this static characterization, e.g., on the support, can be used to constrain or guide time-resolved 3D-POT with just four photon energies. At the same time, this initial measurement provides a precise characterization of the background that can be harnessed in subsequent measurements. This leads to a tremendous reduction of the total required data collection and aids in the identification of weak excited-state signals, thereby supporting the recording of photon energy-dependent dynamics. Taking the current exposure time of 2 h per photon energy as an example, we can see that 4 photon energies ×10 pump-probe delay steps would yield a total measurement of 80 h that is comparable to other momentum microscopy studies (e.g., ref. ^6^, ≈70 h). Similarly, for studies of hybridization in, e.g., organic–inorganic heterostructures, an analysis of the available prior knowledge, such as the support and symmetry, can help to identify the minimum sampling in momentum space and thus the minimum required set of photon energies.

In conclusion, we have demonstrated that full 3D-POT can be realized efficiently with a table-top ultrashort HHG-based EUV light source and independently of DFT calculations. We supplemented this setup with tailored orbital reconstruction algorithms, which allow efficient 3D data extraction and may enable ultrafast time-resolved studies in the future. We have achieved full 3D orbital images of the HOMO and LUMO of PTCDA/Ag(110) and found that this can already be achieved using a data set based on only four photon energies, which we recorded over a total timespan of only 8 h. With intrinsic femtosecond time resolution, this result positions 3D-POT as a prime candidate, e.g., for the study of light-matter interaction and exciton dynamics in organic semiconductors^5,6^ or the study of orbital hybridization in organic semiconductor hybrid interfaces^10,23,47^. Finally, improved access to measurements at different photon energies will also help to further develop POT itself, for instance with respect to the plane-wave approximation and final-state effects^48^.

## Methods

### Ultrafast EUV monochromator

The presented photon energy-dependent photoemission measurements were carried out using the Göttingen in-house photoemission setup^25^ in combination with a newly added tunable EUV beamline based on HHG and a pulse-preserving EUV monochromator. The overall setup is shown in Supplementary Fig. 1.

The broadband EUV light is generated by tightly focusing the fundamental of the laser (1030 nm center wavelength, 35 fs pulse duration, 500 kHz repetition rate, 130 *μ*J pulse energy) using a *f* = 7.5 cm lens into an argon jet, yielding photon energies >71 eV. Directly after the HHG source, a combination of a 4 mm aperture and an anti-reflection-coated EUV/IR separation mirror is used to reject most of the fundamental radiation. The Nb_2_O_5_ top coating of the separation mirror allows a high reflection of the EUV radiation, while the dielectric coating ensures an overall IR reflectivity below 0.5%. The actual monochromator consists of two gold-coated toroidal mirrors (*f* = 30 cm, 5° grazing incidence) and a plane grating (5° grazing incidence). The first toroidal mirror collimates the EUV beam, while the second focuses the grating output to a wavelength selection slit. A combined translation/rotation stage allows us to select one of four different plane gratings (100, 300, 400, 600 lines/mm, respectively, see Table 2).

**Table 2:** Overview of the specifications of the used gratings: grazing incidence angle, line density, blaze angle, and operating energy range

	AOI (°)	Line density (lines/mm)	Blaze angle (°)	Energy range (eV)
Grating 1	5	100	2.3	13.2–30.10
Grating 2	5	300	2.8	20.1–51.8
Grating 3	5	400	2.9	47.0–59.0
Grating 4	5	600	2.6	59.0−71.0

The rotation stage is set up such that the center of rotation is on the grating surface, which allows optimal wavelength tuning without the need for realignment. These four different grating configurations allow one to choose the effective spectral resolution, and thereby to minimize temporal broadening while still ensuring the selection of only one high harmonic. The calculated acceptance bandwidth of the 100 *μ*m wavelength selection slit and the expected pulse-front tilt (assuming a worst-case HHG source divergence of 20 mrad) are shown in Supplementary Fig. 2.

After the wavelength-selection slit, we use a single *f* = 80 cm (6° grazing incidence) toroidal mirror to image the monochromator output to the sample stage of our time-of-flight momentum microscope^25^. A 100 nm thin aluminum film is used to reject the remaining fundamental laser light, and a photodiode (AXUV100G, EQ Photonics) on a translation stage is used to measure the EUV flux just before the third toroidal mirror.

We note that the first two toroidal mirrors are mounted horizontally and thus orthogonal to the other optical components of the monochromator (cf. Supplementary Fig. 1). This orientation significantly enhances the overall transmission for *p*-polarized EUV light, which is typically used in ARPES experiments, and leads to a rather polarization-insensitive transmission of the monochromator. See Supplementary Fig. 3 for the predicted efficiency over the full EUV spectrum. In this way, optimal conditions are established for multidimensional ARPES using 25 photon energies spanning from 13 eV to 71 eV.

### Photon energy-dependent momentum microscopy

The PTCDA/Ag(110) sample was prepared in the same way as in ref. ^16^, by sublimation from a Knudsen cell. The Ag(110) substrate was cleaned prior to the sublimation by Ar ion sputtering and thermal annealing, and was kept at room temperature for the sample growth. Low-energy electron diffraction was used to verify the brick-wall PTCDA superstructure.

The presented data were acquired using ten different photon energies between 20.5 and 63.8 eV. We used p-polarized light with a fixed incidence angle of 68° to the surface normal. At each photon energy, we measured a three-dimensional data set depending on the kinetic energy and both parallel momenta of the photoemitted electrons (cf. ref. ^25^). Before further analysis, the data were calibrated in energy and momentum and corrected for distortion. To this end, the data for different photon energies were first aligned in momentum based on their cross-correlation. Second, the momentum calibration and distortion were determined by fitting the photoemission horizon of the data set measured with *hν* = 20.5 eV with an ellipse and subsequently mapping this ellipse onto a circle with a fixed radius $0.514\sqrt{{E}_{{{\rm{kin}}}}}$ for each data slice with kinetic energy *E*_kin_. Observing that the distortion did not noticeably change over the full measurement duration (i.e., between photon energies), the same parameters were used to calibrate and correct all acquired data sets.

The intensity calibration measurements were performed separately from the longer integrations used to extract momentum maps. For each photon energy, we measured the incident EUV flux before and after a short (5 min) integration of the momentum-integrated photoelectron spectrum. To eliminate contributions from residual IR light, we measured the diode photocurrent with and without Ar gas in the HHG chamber, i.e., with and without EUV light. The photodiode position was optimized for each photon energy, and the EUV flux was determined from the average of the before-and-after measurements.

A complicating factor in the intensity calibration is the photon-energy-dependent illumination profile on the sample, which typically exceeded the measurement area defined by the real-space aperture in the momentum microscope. Therefore, the fraction of all EUV photons (as measured with the photodiode) incident on the measurement area was determined from two real-space photoemission microscopy images acquired with and without the real-space aperture. Subsequently, the momentum microscope was switched to momentum mode to record the photoelectron spectrum under conditions identical to those used for the longer momentum-map integrations. Finally, the photon-energy-dependent photoemission cross-section was calculated by relating the EUV photon flux with the photoemission current, which was determined by a Gaussian fit of the HOMO.

To determine the energy resolution of each photoemission data set, the spectrum was fitted by the Fermi–Dirac distribution accounting for the thermal broadening at *T* = 300 K. The momentum resolution was determined by fitting an error function at the photoemission horizon of the momentum map of the HOMO. For photon energies higher than 50 eV, the photoemission horizon was no longer measured due to clipping inside the momentum microscope. The relevant parameters for each measured photon energy (i.e., used grating, energy- and momentum resolution of the photoemission data set, intensity calibration factor) are summarized in Table 1. Furthermore, the used photon energies for the reconstructions presented in Figs. 3 (7 *hν*) and 4 are also indicated.

### Background subtraction

In order to reconstruct a 3D image of the molecular orbitals from the experimental data, it is necessary to extract two-dimensional momentum fingerprints of the individual orbitals and to subtract background contributions, which can, for example, be due to the *sp* bands of the silver substrate or due to (in)elastically scattered photoelectrons. Here, we present a simple algorithm to extract the molecular orbital fingerprint from each 3D ARPES measurement. The algorithm relies on a simple, but sufficient, modeling of the ARPES spectrum by a Gaussian lineshape of the molecular orbital fingerprint and a background contribution that varies only linearly with the photoelectron kinetic energy. In addition to the description of the algorithm, a Python implementation of the algorithm is published along with the data^49^.

In a first step, we only consider the momentum-integrated photoelectron spectrum *M*(*E*_*b*_). In the binding energy (*E*_*b*_) range from 2.5 to 0 eV, the PTCDA/Ag(110) photoelectron spectrum can be modeled as two (quasi-)Gaussian lines superimposed on a linearly-varying background due to the Ag *sp* bands, modulated by a Fermi–Dirac distribution. Thus,

2 $$M({E}_{b})\approx {\sum }_{O}\frac{1}{{\sigma }_{O}\sqrt{2\pi }}{e}^{-\frac{1}{2}\frac{{({E}_{b}-{\mu }_{O})}^{2}}{{\sigma }_{O}^{2}}}{A}_{O}+\frac{{A}_{B}+{m}_{B}{E}_{b}}{1+{e}^{\frac{{E}_{b}-{\mu }_{B}}{{\sigma }_{B}}}},$$

where the summation over *O* considers both the HOMO and the LUMO, which is occupied due to charge transfer from the Ag surface. The binding energy, line width, and amplitude are given by *μ*_*O*_, *σ*_*O*_, and *A*_*O*_, respectively. The background is described by a constant offset *A*_*B*_, a slope *m*_*B*_, and the Fermi–Dirac distribution width and position *σ*_*B*_ and *μ*_*B*_. For each photon energy, we determine the parameters *μ*_*O*_, *σ*_*O*_, *σ*_*B*_, and *μ*_*B*_ using the lmfit package for Python. These parameters are then fixed for the subsequent momentum-resolved analysis. Note that we observe no photon energy dependence of these parameters, but rather perform the fit for each photon energy to eliminate variations due to differing energy calibrations.

A detailed derivation of the momentum-resolved background subtraction is given in the Supplementary Information, section 2. Typical retrieved momentum-resolved *A*_*O*_, *A*_*B*_, and *m*_*B*_ are shown in Supplementary Fig. 4.

From the full results, we conclude that the linear model of Eq. (2) enables a sufficient subtraction of the background for 3D orbital tomography. Ultimately, we find that this procedure leaves a small homogeneous background, which we attribute to elastically scattered photoelectrons and disorder in the system. We suppress this background using a simple thresholding operation. At some photon energies, a residual signal stemming from the Ag(110) substrate can also be found, which could potentially have an effect on the reconstructions^22^. However, in our case, we find that this background does not noticeably affect the reconstruction quality. An exemplary script to perform this analysis will be published along with this article.

### 3D orbital reconstruction

In order to reconstruct the full 3D molecular orbital from photoemission data recorded at a limited number of photon energies, it is necessary to recover both the unknown phase in the full 3D momentum space and the unknown amplitudes at momenta (*k*) in between the measured hemispheres. Here, we use the cyclic projections (CP) algorithm to find an orbital that simultaneously satisfies the measured data and our prior knowledge, thereby retrieving both the missing amplitude and phase data. Specifically, our prior information includes a loose support of 12 × 18 × 6 Å^3^, roughly matching to the van der Waals size of the molecule, a voxel sparsity (see ref. ^16^), allowing only a finite non-zero number of voxels (800 voxels, i.e., 26% of the support), and the known symmetry of the respective gas-phase molecular orbital (i.e., antisymmetry along the *x*, *y* and *z* axes for the HOMO, and antisymmetry along the *x* and *z* axes but symmetry along the y axis for the LUMO).

Together, the prior information and measurement data yield constraints that define the feasibility space, where the likelihood of an orbital guess is given by the combined discrepancy with all constraints. This is quantified by the reconstruction gap, which is zero for a solution that is consistent with all data and prior knowledge. Starting with a random guess, the CP algorithm seeks to find a solution that minimizes this discrepancy by iteratively enforcing the respective constraints. In this way, CP converges to fixed points (local minima) of the feasibility landscape^50^. However, due to measurement noise and inconsistency between the different constraints, a solution that complies with all given constraints generally does not exist, and additionally, many local minima exist^27^. Therefore, as discussed in the section “Accuracy, reliability, and experimental design for time-resolved 3D-POT”, we run many independently initialized reconstruction trials and assess their quality based on the reconstruction gap.

### Clustering of the reconstruction results

To evaluate the reliability of the reconstruction algorithm, we performed multiple reconstructions for the same parameter set starting from 100 random initializations. To acquire an overview of the resulting data and specifically to extract typical, commonly occurring reconstructed orbitals, we apply a clustering algorithm based on a principal component analysis (PCA) and the DBSCAN algorithm. In a first step, we transform the data using PCA, keeping all components so that no information is lost. Subsequently, we apply DBSCAN density-based spatial clustering (as implemented in the scikit-learn package for Python), where the minimum cluster size was set to 6 and the maximal distance between neighboring samples within one cluster was set to 0.012 (0.018) for the HOMO (LUMO). The script used for this analysis is published along with the data^49^.

## Supplementary information

Supplementary Information

Transparent Peer Review file

## Data Availability

The experimental data and reconstructed orbitals that support the findings of this study are available from GRO. Data under the identifier 10.25625/1EMFFL^49^.

## Code availability

The code to extract orbital momentum maps from POT data at a single photon energy is available from GRO.Data^49^. The code to reconstruct 3D orbitals from the 3D-POT data is available from the Gitlab repository https://gitlab.gwdg.de/nam/ProxPython/-/tree/orbit3D_realData under a Creative Commons BY 4.0 license.

## References

[1] Puschnig, P. et al. Reconstruction of molecular orbital densities from photoemission data. *Science* **326**, 702–706 (2009). 19745118 10.1126/science.1176105

[2] Wallauer, R. et al. Tracing orbital images on ultrafast time scales. *Science* **371**, 1056–1059 (2021). 33602865 10.1126/science.abf3286

[3] Adamkiewicz, A. et al. Coherent and incoherent excitation pathways in time-resolved photoemission orbital tomography of CuPc/Cu(001)-2O. *J. Phys. Chem. C* **127**, 20411–20418 (2023).

[4] Baumgärtner, K. et al. Ultrafast orbital tomography of a pentacene film using time-resolved momentum microscopy at a FEL. *Nat. Commun.* **13**, 2741 (2022). 35585096 10.1038/s41467-022-30404-6 PMC9117673

[5] Neef, A. et al. Orbital-resolved observation of singlet fission. *Nature* **616**, 275–279 (2023). 37045918 10.1038/s41586-023-05814-1 PMC10097594

[6] Bennecke, W. et al. Disentangling the multiorbital contributions of excitons by photoemission exciton tomography. *Nat. Commun.* **15**, 1804 (2024). 38413573 10.1038/s41467-024-45973-x PMC10899218

[7] Schmitt, D. et al. Formation of moiré interlayer excitons in space and time. *Nature* **608**, 499–503 (2022). 35978130 10.1038/s41586-022-04977-7

[8] Madéo, J. et al. Directly visualizing the momentum-forbidden dark excitons and their dynamics in atomically thin semiconductors. *Science* **370**, 1199–1204 (2020). 33273099 10.1126/science.aba1029

[9] Dong, S. et al. Direct measurement of key exciton properties: Energy, dynamics, and spatial distribution of the wave function. *Nat. Sci.* **1**, e10010 (2021).

[10] Bennecke, W. et al. Hybrid Frenkel–Wannier excitons facilitate ultrafast energy transfer at a 2D-organic interface. *Nat. Phys.* **21**, 1973–1980 (2025). 10.1038/s41567-025-03075-5 PMC12695656 41393305

[11] Yang, X. et al. Identifying surface reaction intermediates with photoemission tomography. *Nat. Commun.* **10**, 3189 (2019). 31320632 10.1038/s41467-019-11133-9 PMC6639300

[12] Hurdax, P. et al. Large distortion of fused aromatics on dielectric interlayers quantified by photoemission orbital tomography. *ACS Nano* **16**, 17435–17443 (2022). 36239301 10.1021/acsnano.2c08631 PMC9620409

[13] Yang, X. et al. Momentum-selective orbital hybridisation. *Nat. Commun.* **13**, 5148 (2022). 36055995 10.1038/s41467-022-32643-z PMC9440066

[14] Lüftner, D. et al. Imaging the wave functions of adsorbed molecules. *Proc. Natl Acad. Sci. USA* **111**, 605–610 (2014). 24344291 10.1073/pnas.1315716110 PMC3896198

[15] Kliuiev, P., Latychevskaia, T., Osterwalder, J., Hengsberger, M. & Castiglioni, L. Application of iterative phase-retrieval algorithms to ARPES orbital tomography. *New J. Phys.* **18**, 093041 (2016).

[16] Jansen, M. et al. Efficient orbital imaging based on ultrafast momentum microscopy and sparsity-driven phase retrieval. *New J. Phys*. **22**, 063012 (2020).

[17] Repp, J., Meyer, G., Stojković, S. M., Gourdon, A. & Joachim, C. Molecules on insulating films: scanning-tunneling microscopy imaging of individual molecular orbitals. *Phys. Rev. Lett.* **94**, 026803 (2005). 15698209 10.1103/PhysRevLett.94.026803

[18] Cocker, T. L., Peller, D., Yu, P., Repp, J. & Huber, R. Tracking the ultrafast motion of a single molecule by femtosecond orbital imaging. *Nature* **539**, 263–267 (2016). 27830788 10.1038/nature19816 PMC5597038

[19] Itatani, J. et al. Tomographic imaging of molecular orbitals. *Nature* **432**, 867–871 (2004). 15602553 10.1038/nature03183

[20] Vozzi, C. et al. Generalized molecular orbital tomography. *Nat. Phys.* **7**, 822–826 (2011).

[21] Weiß, S. et al. Exploring three-dimensional orbital imaging with energy-dependent photoemission tomography. *Nat. Commun.* **6**, 8287 (2015). 26437297 10.1038/ncomms9287 PMC4600719

[22] Graus, M. et al. Three-dimensional tomographic imaging of molecular orbitals by photoelectron momentum microscopy. *Eur. Phys. J. B* **92**, 80 (2019).

[23] Krumland, J. & Cocchi, C. Ab initio modeling of mixed-dimensional heterostructures: a path forward. *J. Phys. Chem. Lett.* **15**, 5350–5358 (2024). 38728611 10.1021/acs.jpclett.4c00803 PMC11129309

[24] Baumgärtner, K. et al. Femtosecond concerted rotation of molecules on a 2D material interface. *Nat. Commun.* **17**, 2110 (2026). 41760610 10.1038/s41467-026-69801-6 PMC12954116

[25] Keunecke, M. et al. Time-resolved momentum microscopy with a 1 MHz high-harmonic extreme ultraviolet beamline. *Rev. Sci. Instrum.* **91**, 063905 (2020). 32611056 10.1063/5.0006531

[26] Frassetto, F. et al. Single-grating monochromator for extreme-ultraviolet ultrashort pulses. *Opt. Express* **19**, 19169–19181 (2011). 21996859 10.1364/OE.19.019169

[27] Dinh, T. L., Jansen, G. S. M., Luke, D. R., Bennecke, W. & Mathias, S. A minimalist approach to 3D photoemission orbital tomography: algorithms and data requirements. *New J. Phys.* **26**, 043024 (2024).

[28] Gadzuk, J. W. Surface molecules and chemisorption. II. Photoemission angular distributions. *Phys. Rev. B* **10**, 5030–5044 (1974).

[29] Krausz, F. & Ivanov, M. Attosecond physics. *Rev. Mod. Phys.* **81**, 163 (2009).

[30] Mathias, S. et al. Angle-resolved photoemission spectroscopy with a femtosecond high harmonic light source using a two-dimensional imaging electron analyzer. *Rev. Sci. Instrum.* **78**, 083105 (2007). 17764311 10.1063/1.2773783

[31] Maklar, J. et al. A quantitative comparison of time-of-flight momentum microscopes and hemispherical analyzers for time- and angle-resolved photoemission spectroscopy experiments. *Rev. Sci. Instrum.* **91**, 123112 (2020). 33379994 10.1063/5.0024493

[32] Kunin, A. et al. Momentum-resolved exciton coupling and valley polarization dynamics in monolayer MoS_2_. *Phys. Rev. Lett.* **130**, 046202 (2023). 36763432 10.1103/PhysRevLett.130.046202

[33] Reutzel, M., Jansen, G. S. M. & Mathias, S. Probing excitons with time-resolved momentum microscopy. *Adv. Phys. X* **9**, 2378722 (2024).

[34] Karni, O., Esin, I. & Dani, K. M. Through the lens of a momentum microscope: viewing light-induced quantum phenomena in 2D materials. *Adv. Mater.* **35**, 2204120 (2023). 10.1002/adma.202204120 35817468

[35] Heber, M. et al. Multispectral time-resolved energy-momentum microscopy using high-harmonic extreme ultraviolet radiation. *Rev. Sci. Instrum.* **93**, 083905 (2022). 36050085 10.1063/5.0091003

[36] Grazioli, C. et al. CITIUS: an infrared-extreme ultraviolet light source for fundamental and applied ultrafast science. *Rev. Sci. Instrum.* **85**, 023104 (2014). 24593346 10.1063/1.4864298

[37] Poletto, L., Frassetto, F. & Villoresi, P. Ultrafast grating instruments in the extreme ultraviolet. *IEEE J. Sel. Top. Quantum Electron.* **18**, 467–478 (2012).

[38] Wießner, M. et al. Electronic and geometric structure of the PTCDA/Ag(110) interface probed by angle-resolved photoemission. *Phys. Rev. B* **86**, 045417 (2012).

[39] Puschnig, P. *Molecular Orbital Database* http://physikmdb.uni-graz.at:5001/ (accessed on 11 June 2026).

[40] Dauth, M. et al. Angle resolved photoemission from organic semiconductors: Orbital imaging beyond the molecular orbital interpretation. *New J. Phys.* **16**, 103005 (2014).

[41] Krylov, A. I. From orbitals to observables and back. *J. Chem. Phys.* **153**, 080901 (2020). 32872858 10.1063/5.0018597

[42] Mercurio, G. et al. Adsorption height determination of nonequivalent C and O species of PTCDA on Ag(110) using X-ray standing waves. *Phys. Rev. B* **87**, 045421 (2013).

[43] Luke, D. R. Relaxed averaged alternating reflections for diffraction imaging. *Inverse Probl.* **21**, 37–50 (2004).

[44] Dinh, T. L., Jansen, G. S. M. & Luke, D. R. Cyclic relaxed Douglas–Rachford splitting for inconsistent nonconvex feasibility. *J Optim Theory Appl* **209**, 50 (2026).

[45] Dinh, T. L., Bennecke, W., Jansen, G. S. M., Luke, D. R. & Mathias, S. Algorithmic approaches to avoiding bad local minima in nonconvex inconsistent feasibility. Preprint at https://arxiv.org/abs/2502.19052 (2025).

[46] Caruso, F. et al. The 2025 roadmap to ultrafast dynamics: frontiers of theoretical and computational modelling. Preprint at https://arxiv.org/abs/2501.06752 (2025).

[47] Gonzalez Oliva, I., Caruso, F., Pavone, P. & Draxl, C. Hybrid excitations at the interface between a MoS_2_ monolayer and organic molecules: a first-principles study. *Phys. Rev. Mater.* **6**, 054004 (2022).

[48] Kern, C. S. et al. Simple extension of the plane-wave final state in photoemission: bringing understanding to the photon-energy dependence of two-dimensional materials. *Phys. Rev. Res.* **5**, 033075 (2023).

[49] Bennecke, W. et al. Replication data for: table-top three-dimensional photoemission orbital tomography with a femtosecond extreme ultraviolet light source. *GRO.data*. 10.25625/1EMFFL (2025). 10.1038/s41467-026-74308-1 PMC13282482 42321168

[50] Luke, D. R., Thao, N. H. & Tam, M. K. Quantitative convergence analysis of iterated expansive, set-valued mappings. *Math. Oper. Res.* **43**, 1143–1176 (2018).

