# Supplementary material for: Table-top three-dimensional photoemission orbital tomography with a femtosecond extreme ultraviolet light source
Source: Nat Commun. 2026 Jun 19;17:5457. doi: 10.1038/s41467-026-74308-1 (PMC13282482; doi:10.1038/s41467-026-74308-1)
Supplement: Supplementary file 1 — Supplementary Information [file 41467_2026_74308_MOESM1_ESM.pdf]

Supplementary Information: Table-top three-dimensional photoemission orbital tomography with a femtosecond extreme ultraviolet light source

Wiebke Bennecke[1], Thi Lan Dinh[2], Jan Philipp Bange[1], David Schmitt[1], Marco Merboldt[1], Lennart Weinhagen[1], Bent van Wingerden[1], Fabio Frassetto[3], Luca Poletto[3], Marcel Reutzel[1], Daniel Steil[1], D Russell Luke[2], Stefan Mathias[1,4,*] and G S Matthijs Jansen[1,*]

[1] 1st Institute of Physics, University of Göttingen, Friedrich-Hund-Platz 1, 37077, Göttingen, Germany

[2] Institute for Numerical and Applied Mathematics, University of Göttingen, Lotzestrasse 16-18, 37083 Göttingen , Germany

[3] Institute for Photonics and Nanotechnologies CNR-IFN, 35131 Padova, Italy

[4] International Center for Advanced Studies of Energy Conversion (ICASEC), University of Göttingen, Göttingen, Germany

E-mail: gsmjansen@uni-goettingen.de

E-mail: smathias@uni-goettingen.de

Abstract. The supplementary information includes a schematic figure of the extreme ultraviolet monochromator and figures on the energy resolution, temporal broadening and overall transmission of the monochromator. Concerning the background subtraction routine, a description of the algorithm and a figure with exemplary background subtraction results are given. Furthermore, additional details on the orbital reconstruction are given, including reconstructions with a tighter support and reconstruction of the formerly lowest unoccupied molecular orbital (LUMO) is given. Finally, a discussion of the momentum-space fingerprint of the two most common HOMO and LUMO reconstructions is given.

1. Experimental setup

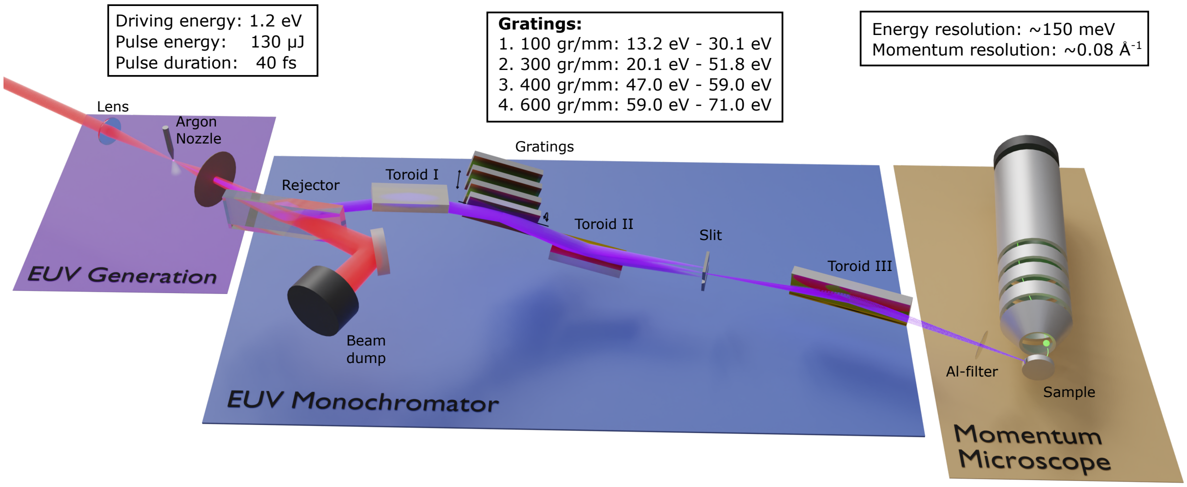

Supplementary Fig. 1. Schematic overview of the ultrafast photon-energy-dependent momentum microscopy setup. On the left, coherent broadband EUV light is generated using 1.2 eV laser pulses focused with $f = 7.5$ cm focal length into an argon gas jet. At an anti-reflection-coated mirror for IR, the EUV light is reflected into the monochromator. This consists of 2 toroidal mirrors (operating at 5° grazing incidence) and a set of interchangeable and rotatable gratings mounted in the off-plane diffraction geometry. For each desired photon energy, the least dispersive grating that fully isolates the selected harmonic is chosen to limit the effect of spatial chirp on the pulse duration. The toroidal mirrors I and II collimate and refocus the monochromatized light into a wavelength-selection slit, while a third, long-distance toroidal mirror (III) refocuses the transmitted EUV light to the photoemission momentum microscope. Finally, an EUV-sensitive photodiode can be moved into the beamline just before the last toroidal mirror to measure the monochromatized EUV flux. Specific details on the gratings are given in Methods, section 8.1.

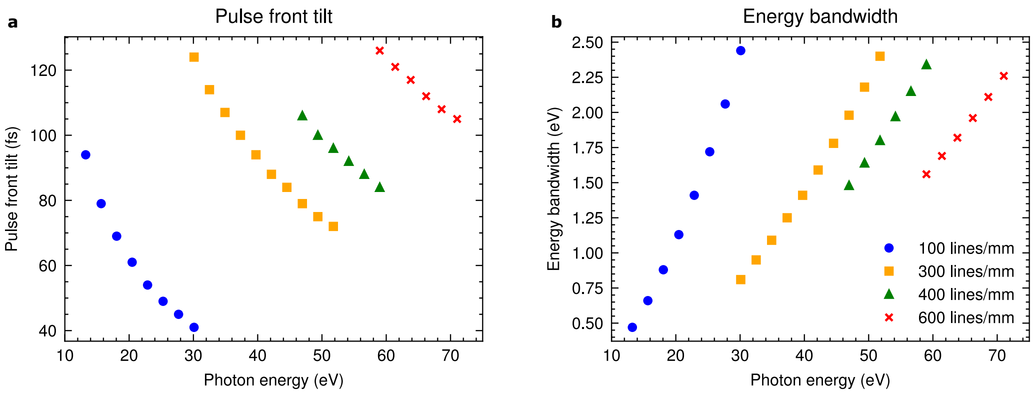

Supplementary Fig. 2. a Temporal broadening due to the pulse front tilt and **b** energy acceptance bandwidth ΔE at the 100 μm slit. The individual high harmonics can be separated when $\Delta E < 2.4$ eV.

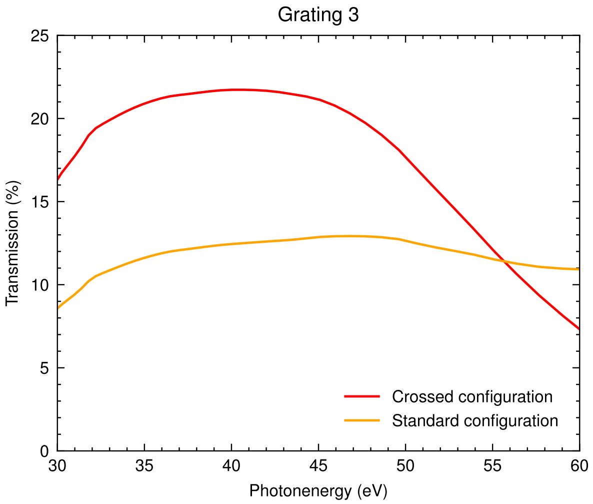

Supplementary Fig. 3. Calculated overall transmission of the EUV monochromator. For simplicity, the initial grazing incidence plate was not included in the calculation, and the grating line spacing was set to 400 lines/mm. The chosen 'crossed' configuration, i.e. with two toroidal mirrors mounted horizontally, results in an overall higher transmission for p-polarized light compared to the standard configuration (all optics mounted vertically). Additionally, the 'crossed' configuration reduces the polarization dependence of the transmission. The efficiency was calculated using the optical constants of gold, while the grating efficiency was calculated using the Gsolver software. Surface roughness was ignored in the calculation.

2. Momentum-resolved background subtraction algorithm

The momentum-integrated analysis of the photoelectron spectrum is described in the Methods, section 7.3. A typical result is shown in Supplementary Fig. S4a.

For the momentum-resolved analysis, we now consider that A_O and A_B are each momentum-dependent intensity distributions, i.e., $A_O, A_B \in \mathbb{R}_+^{N_x \times N_y}$, where N_x and N_y are the number of voxels along the k_x and k_y axes, respectively. Also m_B is momentum dependent, but takes both positive and negative values. Since this simple description of the background cannot account for the strongly dispersive band structure of the *sp* bands observed in this energy region, we constrain the analysis to a small energy region centered on the molecular fingerprint of interest. To extract the HOMO fingerprint, we therefore consider only binding energies between approximately 2.5 and 1.4 eV, while for the LUMO we will consider energies between 1.4 and 0 eV.

Let $M = (M_1, \ldots, M_{N_z})$ be a vector where each entry $M_i \in \mathbb{R}^{N_x \times N_y}$ represents the intensity distribution (momentum map) recorded at the ith binding energy. N_z indicates the number of binding energies. Similarly, let $E = (E_1, \ldots, E_{N_z})$ indicate the corresponding (real-valued) energies. We then rewrite (2) as:

$$M_i \approx \eta_O^{(i)} A_O + \eta_B^{(i)} A_B + \zeta_B^{(i)} m_B, \;\; \forall i = 1, \ldots, N_z, \tag{1}$$

where $A_O, A_B \in \mathbb{R}_+^{N_x \times N_y}$, $m_B \in \mathbb{R}^{N_x \times N_y}$, and

$$\eta_O^{(i)} = \frac{1}{\sigma_O \sqrt{2\pi}} e^{-\frac{1}{2}\frac{(E_i - \mu_O)^2}{\sigma_O^2}}, \quad \eta_B^{(i)} = (1 + e^{\frac{E_i - \mu_B}{\sigma_B}})^{-1}, \quad \zeta_B^{(i)} = E_i \eta_B^{(i)}. \tag{2}$$

We can then define the objective function g, acting on a guess of the momentum-dependent distributions $X = (A_O, A_B, m_B)$ as $g(X) = (g_1(X), \ldots, g_{N_z}(X))$ with

$$g_i(X) = \eta_O^{(i)} A_O + \eta_B^{(i)} A_B + \zeta_B^{(i)} m_B - M_i, \;\; \forall i = 1, \ldots, N_z. \tag{3}$$

The absolute squared approximation error is then given by $f(X) = \|g(X)\|^2 = \sum_{i=1}^{N_z} \|g_i(X)\|^2$. To extract the momentum fingerprints from the data, we therefore consider the minimization problem

$$\min_{X \in \Omega} \quad f(X), \tag{4}$$

where the domain Ω incorporates the non-negativity constraint on A_O, A_B and reality constraint on m_B. Note that problem (4) is convex since the objective function f and the domain Ω are convex. Therefore, 4 attains only a single minimum value.

We have used the Projected Gradient method to solve (4). The algorithm is implemented as follows: Given an initial point $X^{(0)} \in (\mathbb{R}^{N_x \times N_y})^3$ (e.g., $X = \mathbf{0}$), $s > 0$ small enough, for $n = 1, 2, \ldots,$ do

$$X^{(n+1)} = P_\Omega \left(X^{(n)} - s \nabla f(X^{(n)}) \right). \tag{5}$$

Then, by setting a suitable step size s, the sequence $(X^{(n)})_{n=1}^{\infty}$ converges to an optimizer $X^\star$ of problem (4), see [1].

We now compute ∇f and P_Ω. Set $f_i(X) = \|g_i(X)\|^2$ for all $i = 1, \ldots, N_z$. Then we have $f(X) = \sum_{i=1}^{N_z} f_i(X)$, and therefore $\nabla f(X) = \sum_{i=1}^{N_z} \nabla f_i(X)$ with

$$\nabla f_i(X) = 2 \begin{pmatrix} \frac{\partial g_i}{\partial A_O}(X) \cdot g_i(X) \\ \frac{\partial g_i}{\partial A_B}(X) \cdot g_i(X) \\ \frac{\partial g_i}{\partial m_B}(X) \cdot g_i(X) \end{pmatrix} = 2 \begin{pmatrix} \eta_O^{(i)} \mathbb{1}_{N_x \times N_y} \cdot g_i(X) \\ \eta_B^{(i)} \mathbb{1}_{N_x \times N_y} \cdot g_i(X) \\ \zeta_B^{(i)} \mathbb{1}_{N_x \times N_y} \cdot g_i(X) \end{pmatrix} \quad \forall i = 1, \ldots, N_z. \tag{6}$$

Supplementary Fig. 4b shows typical results retrieved by this procedure.

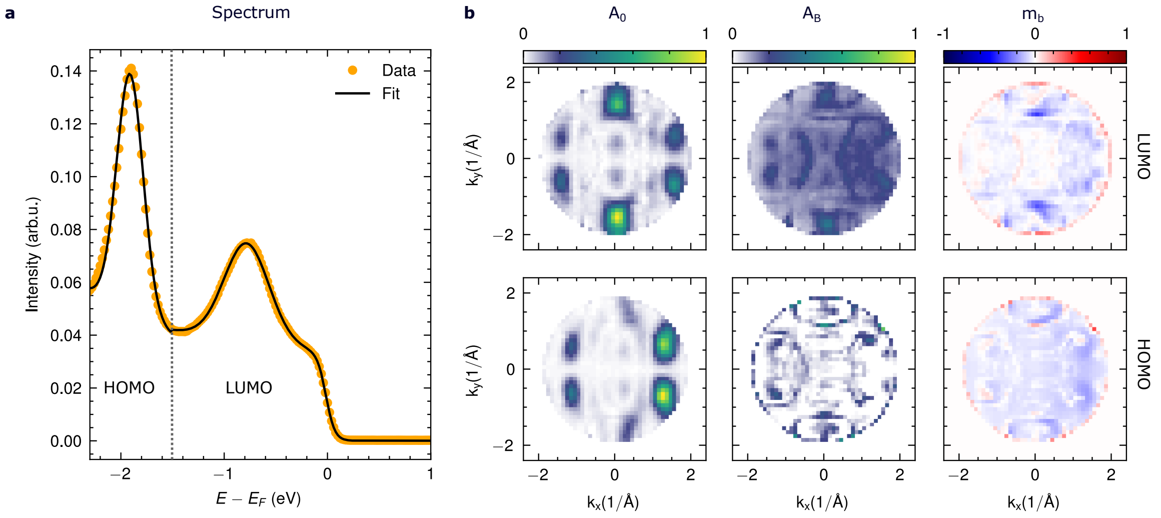

Supplementary Fig. 4. Background subtraction for $h\nu = 22.5$ eV. a Fitted momentum-integrated spectrum according to Eq. (2) (see Methods). The LUMO and HOMO peak was fitted separately. The corresponding energy ranges are indicated by the dotted gray line. **b** Amplitude of the Gaussian peak (A_0), the linear background amplitude (A_B) and the corresponding slope (m_b) for the LUMO (top row) and HOMO (bottom row).

3. Tight support analysis

The support constraint ($12 \times 18 \times 6$ Å^3) for the orbital reconstruction presented in the main text was chosen to match the van-der-Waals size of the molecule. Here, we discuss reconstruction results for a tighter support constraint ($10.5 \times 16.5 \times 4.5$ Å^3). This increases the reliability of the reconstruction and yields reliable results for the data set consisting of four distinct photon energies. In analogy to Fig. 4, Supplementary Fig. 5 shows the gap distribution and clustering results based on the reconstruction using the tighter support (see Methods section 7.5 for details on the clustering).

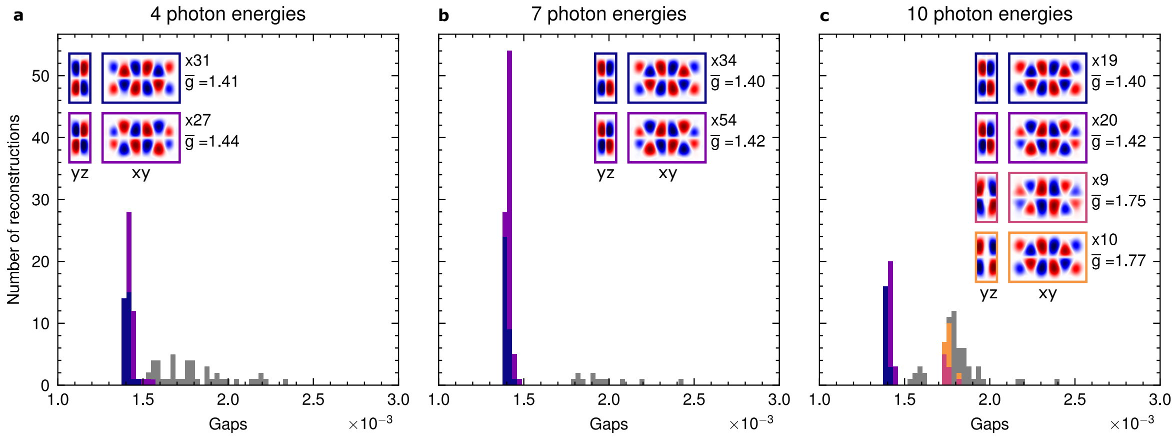

Supplementary Fig. 5. Comparison of the orbital reconstruction results of the HOMO for 4, 7 and 10 photon energies based on a smaller support size ($10.5 \times 16.5 \times 4.5$ Å^3). For each data set, the gap of 100 independent reconstructions was analyzed, and DBSCAN clustering (see Methods) was used to determine the most prominent typical recovered orbitals. From top to bottom, the insets show vertical (yz) and horizontal (xy) slices through the averaged orbital (i.e., the cluster center) of the retrieved clusters, the cluster size, and ($10^3\times$) the average gap ($\bar{g}$). The colored boxes correspond to the applied support constraint. The gaps of the corresponding reconstructions are colored accordingly in the histogram. Reconstructions which could not be assigned to a cluster are colored gray. In contrast to the van der Waals support, the reconstruction based on four different photon energies **a** leads to a well-defined global minimum with two solutions which differ by a phase flip in momentum space (cf. Supplementary Section 5). The same applies for the data sets consisting of seven **b** and ten **c** distinct photon energies, where the number of unassigned reconstructions (gray) is reduced compared to the loose support van der Waals support (cf. Fig. 4).

4. LUMO analysis

Analogous to the reconstruction of the HOMO in the main manuscript, we use the cyclic projection algorithm [2] with identical parameters to reconstruct the LUMO from the respective momentum maps (cf. Fig. 2). We have run 100 independent reconstructions starting from random initializations using 4, 7 and 10 different photon energies (cf. Table 2). As for the HOMO, we find seven photon energies to strike a good balance between reconstruction reliability and measurement time. Fig. 6 shows an exemplary reconstruction in momentum- and real-space based on the sparse measured momentum data. The data are compared to the corresponding KS orbital of the gas-phase molecule calculated by DFT (extracted from Ref. [3]) with the low-pass filter in place. We find excellent agreement between the in-plane (x, y) and out-of-plane (z) distribution, with minor differences that we attribute to increased background contributions in the momentum maps [4].

In order to investigate the reliability of the reconstruction based on 4, 7, and 10 distinct photon energies, Fig. 7 shows the histogram of the reconstruction gaps. Additionally, the data was clustered using principal component analysis and DBSCAN

as described in the Methods section 7.5. Generally, we observe very similar behavior as for the HOMO. Firstly, independent of the used photon energies, we find two most likely reconstructions that can be traced back to a sign change of the phase. Secondly, the gaps consistently increase with the number of photon energies. Thirdly, the reconstruction using seven distinct photon energies yields the most reliable results with the fewest numbers of clusters. These observed trends can be rationalized by similar reasons as those outlined for HOMO in the main text.

5. Momentum space analysis of the most likely reconstructions

The employed reconstruction algorithms yields two most likely reconstructions of the HOMO and LUMO (shown in Fig. 4 and Supplementary Fig. 7, respectively). Supplementary Fig. 8 shows a cut through the real and momentum space representations of the corresponding estimated orbitals. For both the HOMO and the LUMO, a strong difference in the phase profile is accompanied by subtle changes in the amplitude profile, as highlighted by the arrow.

For the HOMO, the first reconstruction cluster (with gap = 1.41) shows a phase flip between the two lobes, as well as a slight slanting of the lobe at $(k_x, k_y) \approx (0.5, 1.5)$ Å^{-1}. On the other hand, the second cluster (gap = 1.42) shows no zero crossing in the amplitude between these lobes. Comparison with Fig. 2 thus suggests a better match between the reconstruction and the measurement data for the first cluster. For the LUMO, the first cluster (gap = 1.42) shows a weak, opposite-phase lobe that inserts between the stronger photoemission features, and also two elongations of the main (upper) lobe curving towards the center are seen. For the second cluster (gap = 1.42), only the zero-crossings and lobes show a significantly different shape. Comparison with Fig. 2 (particularly for 20.5 eV), supports the identification of first cluster as the most likely result. These results are also consistent with the expectation from DFT.

6. Comparison of the k_z and z profiles

In Supplementary Fig. 9, the k_z dependence is compared between the measured data, the reconstructed orbital, and the DFT prediction. These profiles were extracted in a small region centered on one of the main photoemission features of each orbital (see box in Supplementary Fig. 9a and d. Concerning the measured data, it is important to note that photoemission momentum maps were first embedded in the full 3D momentum space before a line-out was extracted. In this way, the strong curvature of the constant-energy hemisphere could be accounted for, and multiple k_z values could be extracted from a single momentum map at fixed photon energy.

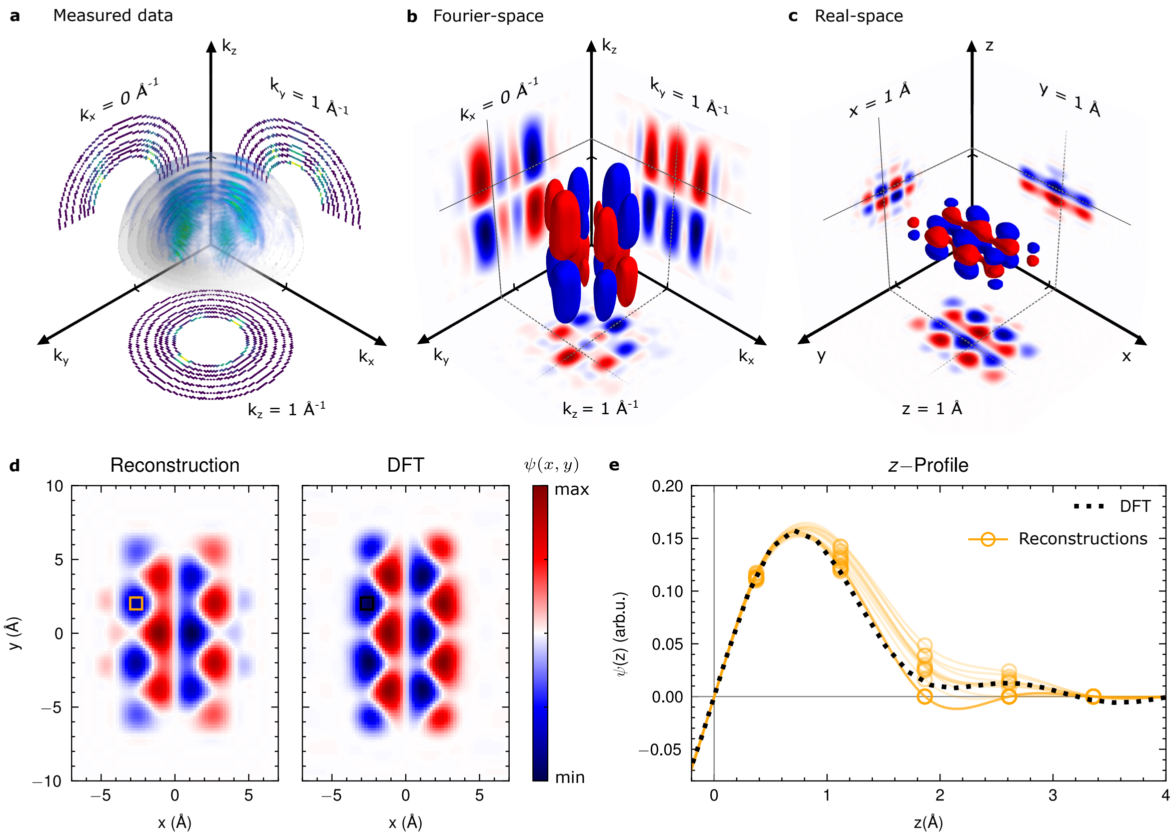

Supplementary Fig. 6. 3D orbital imaging of the PTCDA lowest unoccupied molecular orbital (LUMO) using only seven photon energies. **a** Here, the recorded momentum-space data is visualized on semi-transparent hemispherical shells (see Methods, Table 2 for the exact photon energies). The sparsity of the data is emphasized by slices through the data at $k_{x/y/z} = 1$ Å^{-1}, showing that only a fraction of the voxels in momentum space contain measurement data. **b**,**c** The reconstructed LUMO in momentum space and real space, respectively. Red and blue denote regions of opposite sign. Evidently, the reconstruction algorithm fully recovers the momentum-space amplitudes and phases, and gives a direct view of the full 3D molecular orbital. **d** Comparison of the in-plane (x,y) structure of the reconstructed orbital with the prediction from density functional theory (DFT) of the gas-phase molecule [3]. For an equal comparison, a low-pass filter was applied to the orbital matching to the highest accessible momentum in the experiment. (**e** Comparison of the reconstructed z-dependence of the LUMO (yellow circles; the lines correspond to a Fourier interpolation) with DFT (black dashes), extracted at $(r_x, r_y) = (-2.5, 2)$ Å (see **d**). Since each random initialization of the reconstruction algorithm yields a slightly different orbital, we plot here the z-dependence of 16 independent reconstructions with transparency set to 20% (more saturated yellow thus corresponds to multiple reconstructions yielding the same value). Overall, we observe an excellent agreement between experiment and DFT in both the in-plane structure and the z-dependence, with particularly a good agreement concerning the position of the maximum at $z = 0.8$ Å.

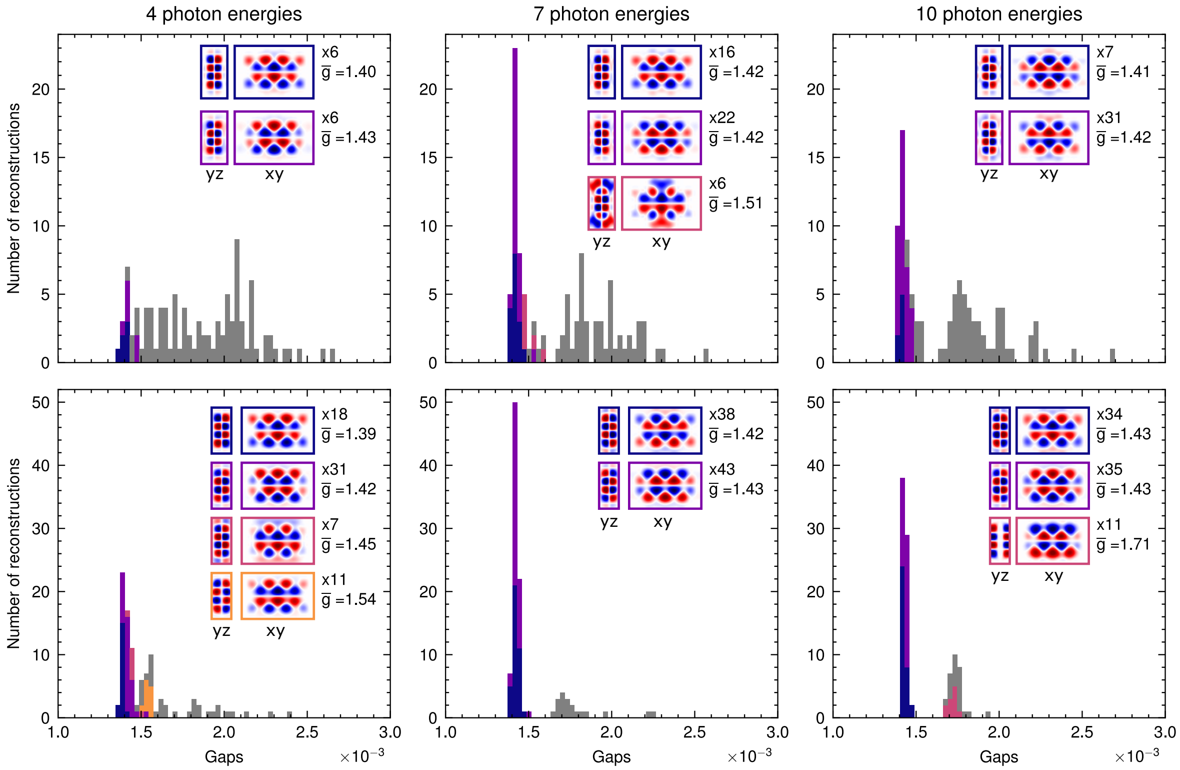

Supplementary Fig. 7. Comparison of the LUMO reconstruction results for 4, 7 and 10 photon energies based on the loose support (a-c) and the tight support (d-f). For each data set, the gap of 100 independent reconstructions was analyzed, and clustering was used to determine the most prominent typical recovered orbitals. Details for the clustering approach are given in the Methods section 8.5. From top to bottom, the insets show vertical (yz) and horizontal (xy) slices through the averaged orbital (i.e., the cluster center) of the retrieved clusters, the cluster size, and ($10^3\times$) the average gap ($\bar{g}$). The colored boxes correspond to the applied support constraint. The gaps of the corresponding reconstructions are colored accordingly in the histogram. Reconstructions which could not be assigned to a cluster are colored gray. The results are comparable to the results obtained for the reconstruction of the HOMO. **a**,**d** When only four photon energies are used for the reconstruction and a loose support ($12 \times 18 \times 6$ Å^3) is applied, the sparse data leads to an increased number of reconstructions which can not be a assigned to a specific cluster and the small gap is not necessarily a good indicator for an accurate reconstruction. The results become more reliable using a tighter support ($10.5 \times 16.5 \times 4.5$ Å^3), where the two most accurate reconstructions (see insets) can be identified by their small gap and only differ by a phase flip in momentum space (cf. Supplementary Section 5). **b**,**e** When seven photon energies are used instead, the global minimum (optimal solution) becomes more defined for the loose support and the tight support restricts the reconstructions almost exclusively to the two most prominent reconstructions (see insets). **c**,**f** Reconstructions based on ten photon energies exhibit very similar behaviour as for seven photon energies. Again the tighter support increases the reliability of the reconstruction.

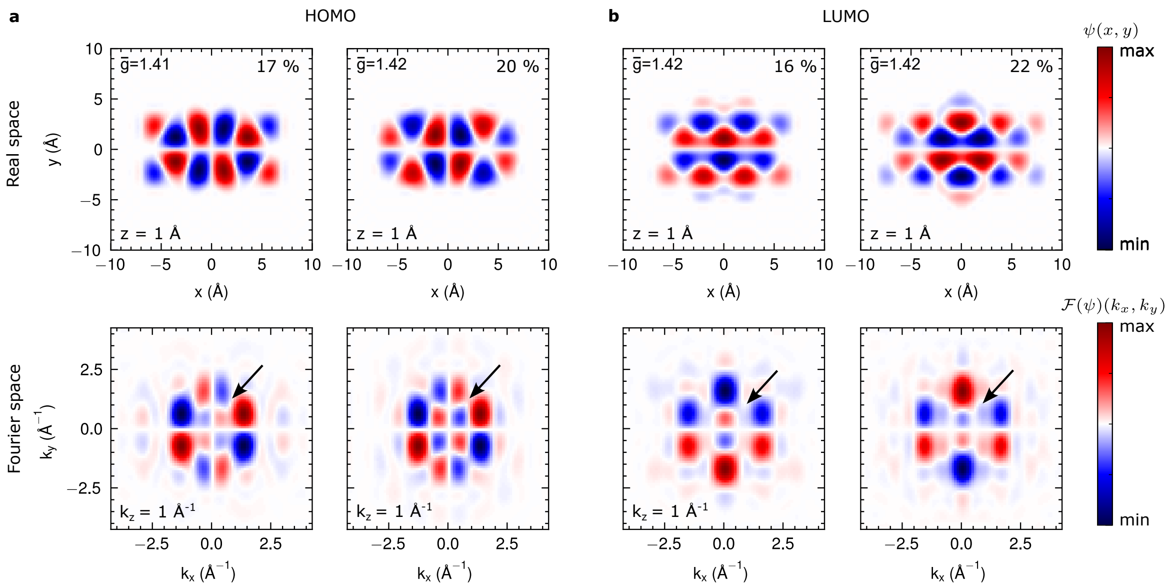

Supplementary Fig. 8. Two most likely reconstructions of the HOMO **a** and the LUMO **b** in real space (top) and momentum space (bottom), each based on the 7-photon energy data set. The area in momentum space where the most notable differences occur is highlighted by an arrow.

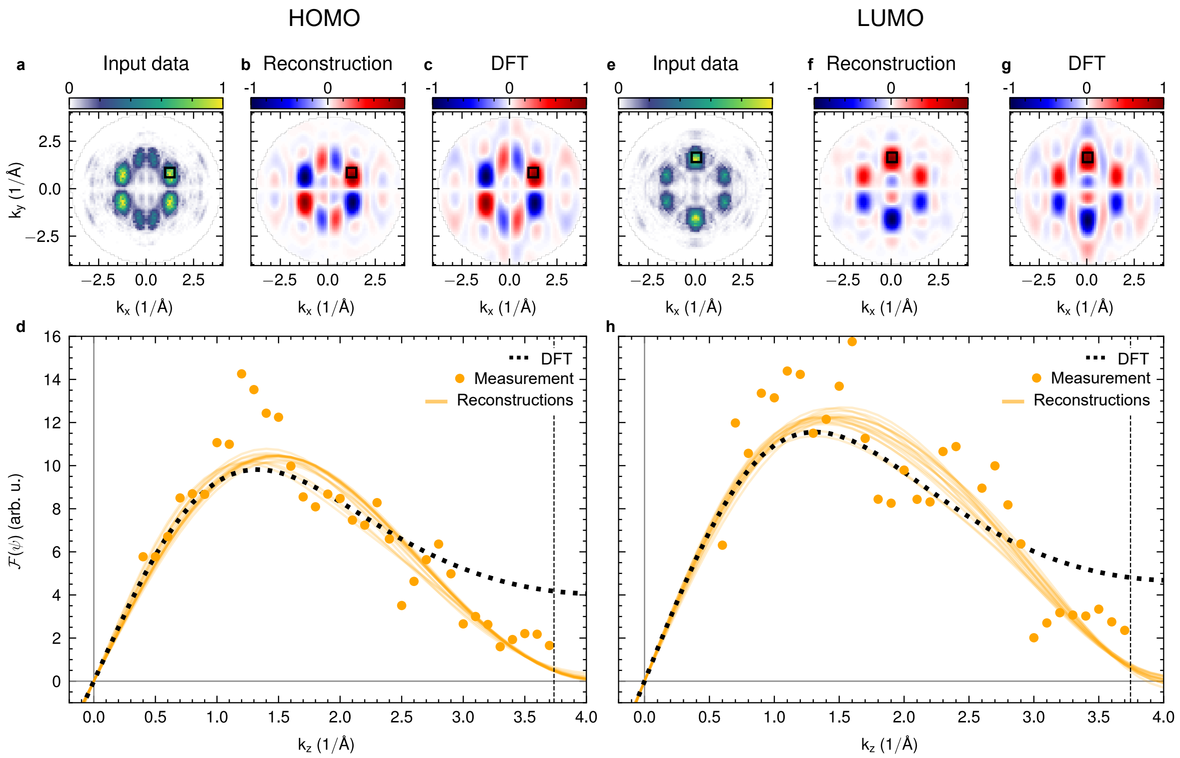

Supplementary Fig. 9. Comparison of the measured and reconstructed k_z profiles with the DFT prediction. The top row shows measured (a,e), reconstructed (b,f) and theoretical (c,g) k_x, k_y maps integrated over $k_z \in [0, \infty)$ for the HOMO (left) and LUMO (right), respectively. The circular features observed in the experimental maps result from the unknown data points between hemispheres. The squares indicate where we extract the k_z profile. d) k_z profile for the HOMO and e) k_z profile for the LUMO. The vertical dashed lines indicate the momentum cutoff, corresponding to $|k| = 4$ Å^{-1}. We attribute the underestimation of the wavefunction amplitude for $k_z \gtrapprox 3.0$ Å^{-1} to a systematic overestimation of the EUV flux due to diffusely scattered low photon-energy EUV light.

Supplementary References

[1] Iusem, A. N. On the convergence properties of the projected gradient method for convex optimization. *Comput. Appl. Math.* **22**, 37–52 (2003).

[2] Dinh, T. L., Jansen, G. S. M., Luke, D. R., Bennecke, W. & Mathias, S. A minimalist approach to 3D photoemission orbital tomography: algorithms and data requirements. *New J. Phys.* **26**, 043024 (2024). URL `https://dx.doi.org/10.1088/1367-2630/ad3e22`.

[3] Puschnig, P. Molecular Orbital Database. URL `http://physikmdb.uni-graz.at:5001/`.

[4] Graus, M. *et al.* Three-dimensional tomographic imaging of molecular orbitals by photoelectron momentum microscopy. *Eur. Phys. J. B* **92**, 80 (2019).
