# Supplementary material for: Table-top three-dimensional photoemission orbital tomography with a femtosecond extreme ultraviolet light source
Source: Nat Commun. 2026 Jun 19;17:5457. doi: 10.1038/s41467-026-74308-1 (PMC13282482; doi:10.1038/s41467-026-74308-1)
Supplement: Supplementary file 2 — Transparent Peer Review file [file 41467_2026_74308_MOESM2_ESM.pdf]

nature portfolio

Peer Review File

Table-top three-dimensional photoemission orbital tomography with a femtosecond extreme ultraviolet light source

Corresponding Author: Dr G. S. Matthijs Jansen

This file contains all reviewer reports in order by version, followed by all author rebuttals in order by version.

Version 0:

Reviewer comments:

Reviewer #1

(Remarks to the Author)
The authors report results from a new approach to spatially reconstruct the wavefunctions of molecular orbitals in three dimensions, based on angle-resolved photoemission (ARPES) data, so-called photoemission orbital tomography (POT). Using an improved design of a previously reported single-grating monochromator (ref. [26] of the manuscript) together with a previously developed algorithm based on cyclic projections (CP) [27] they demonstrate the 3D reconstruction of the frontier orbitals of PTCDA molecules adsorbed on Ag(110) based on ARPES data obtained with a table-top experimental setup.

The authors demonstrate for the first time, that a full 3D reconstruction of molecular orbitals is possible based on a table-top tunable femtosecond EUV source and with a sparse set of ARPES data obtained at only few photon energies - two prerequisites necessary for future time-resolved studies of the evolution of molecular wavefunctions. Although time-resolved data is not presented so far, I believe that the findings of the manuscript will be important for other researchers in the field.

The manuscript is well written and comprehensible. The high-quality experimental data is thoroughly evaluated and the results are adequately presented by the authors. I have only a few questions/remarks:

abstract l.33
absorbed -> adsorbed

p.5 Figs.2c-e
What is the incidence angle of the EUV light? Does the light enter the sample in kx direction? The authors do not explicitly state the used polarization (p-pol.?) of the EUV light. The left-to-right intensity gradients in both the experimental and calculated k-maps are presumably due to the A.k polarization factor? I guess the maps are corrected for the A.k factor prior to application of the CP reconstruction algorithm?

p.7 Fig 3a
The measured data is hardly visible on the three slices through k-space. Maybe, the authors can use a different color coding and/or make the E=const lines thinner.

p.7 Fig 3e
What is the meaning of the open circles? Their z-spacing seems to correspond to the spatial resolution of 0.75 angstrom the authors mention in the text. Is the wavefunction reconstructed only at these points and the solid lines are interpolations? If so, the authors should describe how the interpolation has been performed. Also, interpolation could be performed with a finer z-spacing to get a smoother interpolation curve.

still p.7 Fig 3e
There is a qualitative difference in the z-dependence of the reconstructed wavefunctions and the one calculated with DFT, which the authors do not address in the manuscript. All reconstructions have at least one additional node between 1.5 and 3 angstrom. Especially, at 2.6 angstrom the wavefunction is zero for most reconstructions whereas DFT predicts a maximum. Could this be related to final-state effects in the photoemission process?

p.8ff
Maybe the authors can give a very brief explanation of the CP algorithm. Without knowledge of ref. [27] the reader cannot really understand the meaning of terms like "voxel sparsity", "feasibility landscape" or "reconstruction gap".

p.8 l.201ff
For comparison with their POT results the author use DFT calculated orbitals of planar gas-phase PTCDA. A planar geometry is also assumed as a symmetry constraint in the CP algorithm. However, DFT of PTCDA/Ag(110) as well as experiments with x-ray standing waves suggest that the adsorbed PTCDA molecules adopt a bent geometry with the carboxylic ogygen atoms approx. 0.3 angstrom closer to the metal substrate than the perylene carbon backbone (e.g. Mercurio et al. PRB 87 045421). In their earlier work [27] the authors demonstrated that their method is in principle capable of reconstructing the wavefunction of a bent molecule. It would be interesting to see how the reconstructed orbitals would change if the symmetry constraint perpendicular to the substrate is released. Have the authors performed such calculations? A bent geometry could possibly also explain the slightly different shape of the reconstructed orbitals at the outer lobes compared with gas-phase DFT.

Reviewer #2

(Remarks to the Author)
The ability to perform time resolved studies of molecular orbitals would be of significance to understanding the physics and chemistry of surface processes. The combination presented here of ultrafast momentum microscopy with an algorithm to reconstruct 3D orbitals, that the authors have developed, puts this within reach. Before publication the authors should consider the following.

The summary of the constraints made during the orbital reconstruction on page 8 could be misconstrued to suggest that they have forced/assumed the result – this should be clarified.

P8..“In addition to the experimental data, the algorithm incorporates several constraints on the reconstructed orbital. Namely, we employed the known symmetry in the x, y and z directions, a loose support of 12 × 18 × 6 ˚A (i.e., roughly matching with the van der Waals size of the molecule) and voxel sparsity of the molecular orbital (800 voxels, i.e., 26% of the support).”

I do not believe the authors assumed the known symmetry of the orbital. Due to the single incident geometry of the light an asymmetry in the photoemission distribution results (unlike the original static POT experiments where the photoemission was always recorded in the plane of incidence (and polarization plane) by rotating the sample). The experimental momentum distribution could either be artificially (but reasonably) symmetrised or an A.k factor could be applied which would lead to a symmetric distribution. What have the authors actually done?

From the raw data it seems clear that the incident plane was not in a high symmetry direction- the experimental geometry should be given in the methods section.

For both the HOMO and LUMO two equally probable orbitals are obtained that have different electron density distributions – the authors believe they can identify the correct result:

P10…..” Here, we focus on those reconstructions where the momentum-space phase flips between adjacent lobes, since we estimate that the faulty reconstructions result from residual background noise that obscures the true zero amplitudes between these lobes. Incidentally, this choice is also in line with gas-phase DFT calculations. Taking this into consideration, we emphasize that by analysing the gap and the reconstructed momentum distribution, 3D orbital imaging can be performed without the need to perform additional DFT calculations”.

Are they saying that they choose a result where the sign of the wavefunction changes between emission lobes. This works for the HOMO (in that it yields a result in agreement with the gas DFT orbital) but as I see it (fig S8 and S6) it yields the wrong result for the LUMO as the k-space gas phase DFT orbital does not have a phase change between the lobes.

Reviewer #3

(Remarks to the Author)
In this manuscript, Bennecke et al. perform 3D photoemission orbital tomography (3D-POT) of ordered PTCDA films on Ag(110) using a laser-based XUV source and a time-of-flight momentum microscope. This POT technique uses several major approximations to interpret angle-resolved photoemission (ARPES) as “molecular orbital tomography.” While the approximations give reasonable results for certain cases, for example the large polycyclic aromatic hydrocarbons used in this work, POT is not really a widely applied technique in either ground-state or excited-state (i.e. time-resolved) photoemission. The system studied here, PTCDA on Ag(110), has been extensively studied using both POT (e.g. refs 21 and 22 of the paper) and may other methods.

The main new things we see in the manuscript by Bennecke et al. is A) the use of a femtosecond XUV source, as opposed to a synchrotron, and B) the experimental demonstration of amplitude and phase reconstruction from sparse data using the methods described in ref 27 of the paper. The momentum microscope and 500 kHz HHG source are near the current state-of-the-art in tr-ARPES technology, and the authors do successfully reproduce 3D-POT of the PTCDA/Ag(110) system. The

technical level is very high. However, in our opinion, although the experiments have been done using a femtosecond XUV source, the authors have not really demonstrated the feasibility of time-resolved 3D-POT, A). First of all, there is no time-resolved data in the paper. Second, the demonstrated acquisition times indicate that an excited-state experiment will be very very difficult. The authors state that one ground-state 3D-POT can (barely) be done with 4 photon energies in 8 hours of data acquisition. In excited state measurements, the signal will inherently be much lower. For example, to study dynamics that have any chemical or device relevance for this system, it is likely that the fraction of molecules excited should be 1% or even less to ensure linear dependence on the observed signals on the pump fluence. Thus the signal levels will be at least 100 times smaller, indicating more than 80 hours of acquisition to achieve the same signal levels for just one pump-probe delay. However, it is likely worse than this due to the background being significant, such that it may be that excited-state signal is even below the background. Furthermore, it seems from the results that using only 4 photon energies is likely insufficient to have a faithful reconstruction without additional constraints, inputs, or control experiments (e.g. more photon energies). Overall, we believe that the data presented actually shows that chemically relevant time-resolved 3D-POT using this setup is not feasible, at least with the currently demonstrated signal and background levels. Thus, if this is actually the first realization of B) it may be better to emphasize this more in the paper than A).

Overall, we find this paper a valuable contribution to the field in terms of its systematic approach and clear statements regarding integration times, faithfulness of reconstruction, etc. It is helpful in terms of understanding the signal levels and limitations of the technique. This is difficult experiment, and solid work in an overall difficult and competitive field. We would like to see this work published.

Some more specific comments are below:

1. The authors tried to reconstruct the 3D frontier orbital of PTCDA molecule from the PTCDA grown on Ag(110) surface with brick wall superstructure. They compare with gas-phase PTCDA calculations However, it has been shown that the strong interaction between PTCDA and Ag(110) surface will lead to the formation of chemical bond, which will deviate the LUMO of the adsorbed molecule from free molecule [Physical Review Letters, 2010, 104(23): 233004]. The chemical contribution also bends the molecular plane to favor the interaction of oxygen atoms with the Ag surface atoms underneath [PRB 2013, 87(4): 045421]. Therefore, the interfacial hybridization and molecular bending will complicate the reconstruction from sparse 3D POT data, and the influence of them should be addressed.

2. Related to point 1, it is unclear what is going on in the z direction in figure 3. It seems that in figure 3e, z=0 may correspond to the plane of the atoms (node in the pi orbitals). However, in figure 3c there is no axis labeled z, and the one that is likely z seems not centered on the nodal plane. Furthermore, why is figure 3e not continued to negative z? In reality we would not expect the orbital to be anti-symmetric around z=0 due to the strong impact of the substrate, as discussed in the references of point 1.

3. We do not find any quantitative comparison between the reconstructed k-space data (e.g. fig. 3b) and the measured data (e.g. fig 3a). It is thus difficult to determine how good the reconstruction is.

4. After processing and reconstruction(s), data is compared to Kohn-Sham DFT orbitals. However, strictly speaking these are not rigorously defined. Better is to use the Dyson orbital formalism, which are uniquely defined, as discussed by Krylov [J. Chem. Phys. 153, 080901 (2020)]. At a minimum, the paper should have some discussion of this subtlety, and how the presented orbitals are related to Dyson orbitals. Maybe in Koopman's approximation they are the same in this case using an uncorrelated electronic structure method, but it is not immediately clear.

5. It is not clear what the XUV polarization direction is for the various presented data. It is also not clear how this accounted for in the theory. This could help explain asymmetries in the momentum maps, both in experiment and theory.

6. For the background subtraction, the author has considered the Gaussian distribution of the orbital, the Fermi surface of Ag and some linear background. However, it seems there is still significant background in figures 2d and 2e. This problem has been dealt with previously [The European Physical Journal B, 2019, 92: 1-8], and has been shown to strongly influence reconstructions.

7. In Figure 3e about the z profile, does the yellow circle represent the measured data? If not, could the authors add the measured data after considering the polarization factor, as has been done in previous work [Nature communications, 2015, 6(1): 8287]? Also, the reconstructed data displays a large deviation from the DFT data at about 2 angstroms, could the authors address the reason?

8a. Even with measurements at 10 photon energies, it seems that a spurios phase flip still exists in reconstructed data, so how can one distinguish the best reconstruction between the two phase flip results? If better background subtraction or more measured data is used, could this be avoided?

8b. In terms of item B) mentioned above being potentially the more impactful aspect of the paper, it would be helpful for the authors to comment what level of data quality would be necessary to believe orbital reconstructions without any theoretical orbitals to compare to, if this is actually achievable.

Reviewer #4

(Remarks to the Author)
I co-reviewed this manuscript with one of the reviewers who provided the listed reports. This is part of the Nature Communications initiative to facilitate training in peer review and to provide appropriate recognition for Early Career Researchers who co-review manuscripts.

Version 1:

Reviewer comments:

Reviewer #1

(Remarks to the Author)
By employing a refined photon energy-dependent intensity calibration, the authors present photoemission data of even higher quality, enabling an improved reconstruction of the molecular frontier orbitals. In particular, the reconstructed z-dependence shows better agreement with predictions from DFT, which further strengthens the manuscript's main conclusion - that orbital reconstruction from sparse photoemission data is feasible.

I also appreciate that the authors tried reconstructions without imposing a z-symmetry constraint. It is particularly interesting to observe that the lowest-gap reconstruction of the HOMO appears to capture the slightly bent molecular geometry of PTCDA/Ag(110).

The authors have addressed all of my previous comments satisfactorily in the revised manuscript. Therefore, I recommend publication in its present form.

Reviewer #2

(Remarks to the Author)
The authors have satisfactorially responded to the original report and in doing so have improved the manuscript- I can now recommend it for publication

Reviewer #3

(Remarks to the Author)
The authors have done substantial work here to improve the paper. Overall, it is a very good contribution to the field. The various quantitative comparisons shown give the expert a good sense of the status of the state of the art. This is important work.

Our main concerns regard the effect on the non-expert. We still feel several places in the paper the quality and impact of the results is overstated. While, unfortunately, overstatement is the norm these days in scientific writing, we feel for this subject in particular, it is dangerous. Orbital imaging with photoemission (time-resolved or not) is still very difficult and limited. The paper of Itatani (ref. 19 of this work, also in Nature...), is a prime example of overstatement in the orbital imaging business, and its negative effects are still felt today. “High-harmonic spectroscopy” never amounted to very much, but many people wasted a lot of time on it, and honest practitioners continue to fight the perception that ultrafast (even attosecond) orbital tomography is already a routine thing.

The following changes should be made:

1) The feasibility of time-resolved 3D POT at chemically-relevant excitation densities is at this point a quantitative question the authors can address relatively easily. It the authors want to continue to claim this is “within reach”, they should quantitatively estimate what the integration times and required XUV exposures need to be, given the “optimized” experiment with 2 MHz repetition rate, less background for excited states, etc. They can do this, for example, by running a simulation (e.g. Monte Carlo) with the appropriate signal (e.g. 1% of the current signal level), background, etc. including multiple pump/probe delays. A quantitative estimate is more valuable than the various ‘within reach’ statements. In any event, this would give statements currently in the paper the appropriate context. Example statements include:

Line 74: “In this article, we overcome all of these limitations and demonstrate how 3D-POT can be performed in the laboratory at measurement durations that even permit the application of ultrafast 3D orbital imaging.”

Line 86: “Our results clearly confirm the potential of table-top 3D-POT for systematic studies of orbital hybridization and even for full, femtosecond time-resolved orbital tomography”

Line 233: “promising to study dynamical processes with femtosecond time resolution, too.”

2) Figure S9 is very good, addressing the quality of the reconstruction in momentum space. The figure A1 in the response is also good, addressing our concerns regarding the ability of the reconstruction to discern asymmetry in the z direction. The authors should add A1 to the supplemental material.

3) The ability to reconstruct orbitals without guidance from DFT seems still overstated. The authors should remove the

statement “Thus, we emphasize that by analysing the gap and the reconstructed momentum distribution, 3D orbital imaging can be performed without the need to perform additional DFT calculations.” (line 263).

Reviewer #4

(Remarks to the Author)
I co-reviewed this manuscript with one of the reviewers who provided the listed reports. This is part of the Nature Communications initiative to facilitate training in peer review and to provide appropriate recognition for Early Career Researchers who co-review manuscripts.

Version 2:

Reviewer comments:

Reviewer #3

(Remarks to the Author)
The authors have addressed all our concerns adequately. We recommend the paper for publication.

Reviewer #4

(Remarks to the Author)
I co-reviewed this manuscript with one of the reviewers who provided the listed reports. This is part of the Nature Communications initiative to facilitate training in peer review and to provide appropriate recognition for Early Career Researchers who co-review manuscripts.

Open Access This Peer Review File is licensed under a Creative Commons Attribution 4.0 International License, which permits use, sharing, adaptation, distribution and reproduction in any medium or format, as long as you give appropriate credit to the original author(s) and the source, provide a link to the Creative Commons license, and indicate if changes were made.
In cases where reviewers are anonymous, credit should be given to 'Anonymous Referee' and the source.
The images or other third party material in this Peer Review File are included in the article’s Creative Commons license, unless indicated otherwise in a credit line to the material. If material is not included in the article’s Creative Commons license and your intended use is not permitted by statutory regulation or exceeds the permitted use, you will need to obtain permission directly from the copyright holder.
To view a copy of this license, visit https://creativecommons.org/licenses/by/4.0/

We thank all reviewers and the editor very much for the constructive feedback on our manuscript. In the following, we reply step by step to the comments of the reviewers. A direct response to the reviewers is printed in **blue**, changes to the manuscript are printed in **red**.

General remark

Before responding to the individual reviewers, we first discuss an important advancement that we were able to make based upon the combined feedback. During the revision process, we identified an approach to significantly improve the energy-dependent intensity calibration used in the initial analysis. To implement this improvement, we had to carry out an additional measurement run. Although this caused a delay in submitting the revised version, the resulting improvement clearly justifies the extra effort, and we are confident that the referees will agree. In particular, we implemented the previously omitted photon-energy dependence of the $\vec{A} \cdot \vec{k}$ polarization correction, performed new photon-energy–resolved intensity calibration measurements and did a better subtraction of residual IR light contributions. Doing so allowed us to rescale all momentum maps with more accurate calibration factors, and all affected figures have been updated accordingly. Most notably, this concerns Figures 3 and 4 and Supplementary Figures S5, S6, S7, and S8. It did turn out that these corrections effectively could eliminate artifacts that were present in the preliminary reconstructions (such as spurious nodes in the z-dependence) and moreover drastically improved the reconstructed orbital quality. Note that all conclusions of our original article remain unchanged and are now supported by more robust and higher quality data.

Changes to the manuscript: We have added a discussion on the intensity calibration in Section 7.2 (Methods). The new text reads:

> The intensity calibration measurements were performed separately from the longer integrations used to extract momentum maps. For each photon energy, we measured the incident EUV flux before and after a short (5 minute) integration of the momentum-integrated photoelectron spectrum. To eliminate contributions from residual IR light, we measured the diode photocurrent with and without Ar gas in the HHG chamber, i.e. with and without EUV light. The photodiode position was optimized for each photon energy, and the EUV flux was determined from the average of the before-and-after measurements.
>
> A complicating factor in the intensity calibration is the photon-energy-dependent illumination profile on the sample, which typically exceeded the measurement area defined by the real-space aperture in the momentum microscope. Therefore, the fraction of all EUV photons (as measured with the photodiode) incident on the measurement area was determined from two real-space photoemission microscopy images acquired with and without the real-space aperture. Subsequently, the momentum microscope was switched to momentum mode to record the photoelectron spectrum under conditions identical to those used for the longer momentum-map integrations. Finally, the photon-energy-dependent photoemission cross-section was calculated by relating the EUV photon flux with the photoemission current, which was determined by a Gaussian fit of the HOMO.

Additionally, we have updated all affected figures (Figures 3 and 4, and Supplementary Figures S5, S6, S7, and S8) and Table 2 with the recalibrated data.

Response to Reviewer 1

The authors report results from a new approach to spatially reconstruct the wavefunctions of molecular orbitals in three dimensions, based on angle-resolved photoemission (ARPES) data, so-called photoemission orbital tomography (POT). Using an improved design of a previously reported single-grating monochromator (ref. [26] of the manuscript) together with a previously developed algorithm based on cyclic projections (CP) [27] they demonstrate the 3D reconstruction of the frontier orbitals of PTCDA molecules adsorbed on Ag(110) based on

ARPES data obtained with a table-top experimental setup.

The authors demonstrate for the first time, that a full 3D reconstruction of molecular orbitals is possible based on a table-top tunable femtosecond EUV source and with a sparse set of ARPES data obtained at only few photon energies - two prerequisites necessary for future time-resolved studies of the evolution of molecular wavefunctions. Although time-resolved data is not presented so far, I believe that the findings of the manuscript will be important for other researchers in the field.

The manuscript is well written and comprehensible. The high-quality experimental data is thoroughly evaluated and the results are adequately presented by the authors. I have only a few questions/remarks:

Answer 1.0: We thank the reviewer for their positive review.

Comment 1.1

abstract l.33 absorbed -> adsorbed

Changes to the manuscript 1.1: Thanks for pointing this out, we have corrected it.

Comment 1.2

p.5 Figs.2c-e What is the incidence angle of the EUV light? Does the light enter the sample in kx direction? The authors do not explicitly state the used polarization (p-pol.?) of the EUV light. The left-to-right intensity gradients in both the experimental and calculated k-maps are presumably due to the A.k polarization factor? I guess the maps are corrected for the A.k factor prior to application of the CP reconstruction algorithm?

Answer 1.2: We use p-polarized light entering the sample in x-direction at a fixed incidence angle of 68°. The referee is correct, the observed asymmetry arises from the $|\vec{A}\cdot\vec{k}|^2$ polarization factor. Before the reconstruction, the data are symmetrized according to the PTCDA symmetry (point symmetry), which eliminates most of the effects induced by the polarization factor. Finally, we divide by the symmetrized $\mathbf{A}\cdot\mathbf{k}$ factor to fully correct for its effect.

Changes to the manuscript 1.2:

- Section 3, added

  The observed left-right asymmetry in both experiment and theory stems from the polarization factor $|\vec{A}\cdot\vec{k}|^2$ in equation (1), with the light impinging from the left. To retrieve the momentum space amplitude $|\mathscr{F}(\psi)(\vec{k})|$ required for reconstruction, the data are first symmetrized and subsequently divided by the symmetrized polarization factor.

- Methods Section 7.2, we added "We used p-polarized light with a fixed incidence angle of 68° to the surface normal."

- Related to this, we updated the visualization of $\mathbf{A}\cdot\mathbf{k}$-corrected momentum maps in Figure 2.

Comment 1.3

p.7 Fig 3a The measured data is hardly visible on the three slices through k-space. Maybe, the authors can use a different color coding and/or make the E=const lines thinner.

Answer 1.3: Fig. 3a shows the sparsity of the measured data in the three-dimensional reciprocal space, which is difficult to see by default. Here, the data represented on the hemisphere correspond to the momentum maps

shown in Fig. 2c-e. We agree with the referee that the current illustration might be difficult to understand. To address this issue, we have adapted the caption to better explain what is shown.

Changes to the manuscript 1.3:

- New caption text:

  (a) Here, the raw measurement data is visualized by a cloud of semi-transparent voxels lying on hemispherical shells (see Methods, Table 2 for the exact photon energies). The sparsity of the data is emphasized by slices through the data at $k_{x/y/z} = 1$ Å^{-1}, showing that only a fraction of the voxels in momentum space contain measurement data, whereas most voxels have no data (indicated by white or transparent in the slices and 3D plot, respectively).

Comment 1.4

p.7 Fig 3e What is the meaning of the open circles? Their z-spacing seems to correspond to the spatial resolution of 0.75 angstrom the authors mention in the text. Is the wavefunction reconstructed only at these points and the solid lines are interpolations? If so, the authors should describe how the interpolation has been performed. Also, interpolation could be performed with a finer z-spacing to get a smoother interpolation curve.

Answer 1.4: The reviewer is completely right: the circles are the data points retrieved from the orbital reconstruction, while the solid line is an interpolation. The interpolation was achieved through padding in the Fourier domain, such that the momentum-space data is preserved. The same interpolation is also used for the 2D reconstructed data in Figure 3d, As detailed below, we now make this clear in the manuscript.

Changes to the manuscript 1.4:

- We have added a note to the caption of Fig. 3 to indicate the interpolation: "Comparison of the reconstructed z-dependence of the HOMO (yellow circles; the yellow line shows a Fourier interpolation) with DFT (black dashes), extracted at $(x,y) = (-2,1)$ Å (see d)." We have added the same information to supplementary figure 6.

- We have now improved the plot quality of the interpolation in Fig. 3e by using a spline to smooth out the solid line (i.e., we first use Fourier interpolation, then extract the z profile and finally plot a spline of that data).

Comment 1.5

still p.7 Fig 3e There is a qualitative difference in the z-dependence of the reconstructed wavefunctions and the one calculated with DFT, which the authors do not address in the manuscript. All reconstructions have at least one additional node between 1.5 and 3 angstrom. Especially, at 2.6 angstrom the wavefunction is zero for most reconstructions whereas DFT predicts a maximum. Could this be related to final-state effects in the photoemission process?

Answer 1.5: We agree that the additional node structure in the z-dependence of the reconstructed wavefunctions, which was present in the original submission, required further clarification. As part of the revision, we performed an improved intensity calibration (see General Remark above and Methods, Section 7.2). With this corrected calibration, the situation changes substantially: as shown in the updated Fig. 3, our reconstructions no longer exhibit additional nodes for $z \gtrsim 1$ Å. Instead, the reconstructed wavefunction decays monotonically above the maximum at $z \approx 0.75$ Å, in good agreement with the DFT prediction.

We also observe a correspondingly improved agreement between experiment and theory in the k_z profiles. Consequently, our updated data indicate that final-state effects do not play a dominant role in the PTCDA/Ag(110)

system at the present level of accuracy; the previously observed discrepancies were primarily caused by the imperfect intensity calibration, which has now been corrected.

Changes to the manuscript 1.5:

- As also explained in the General Remark, the z-profiles of both the HOMO and the LUMO were updated in Figures 3 and S6.
- We have also added Supplementary Figure S9, where the measured, reconstructed and predicted k_z profiles are compared. A reference to this figure was added to Section 4: "Additionally, Supplementary Figure S9 provides a quantitative comparison of measured and reconstructed k_z profiles with DFT calculations, demonstrating excellent agreement across all photon energies."

Comment 1.6

p.8ff Maybe the authors can give a very brief explanation of the CP algorithm. Without knowledge of ref. [27] the reader cannot really understand the meaning of terms like "voxel sparsity", "feasibility landscape" or "reconstruction gap".

Answer 1.6: We have followed the suggestion of the reviewer and have described the algorithm now in greater detail in the Method section.

Changes to the manuscript 1.6:

- Section 4, added "A more detailed description of the implemented algorithm can be found in the Method section 7.4."
- Methods, adapted section *7.4. 3D orbital reconstruction*, which now provides more detailed information on the reconstruction algorithm used:

> In order to reconstruct the full 3D molecular orbital from photoemission data recorded at a limited number of photon energies, it is necessary to recover both the unknown phase in the full 3D momentum space and the unknown amplitudes at momenta (k) in between the measured hemispheres. Here, we use the cyclic projection (CP) algorithm to find an orbital that simultaneously satisfies the measured data and our prior knowledge, thereby retrieving both the missing amplitude and phase data. Specifically, our prior information includes a loose support of $12 \times 18 \times 6$ Å^3, roughly matching to the van der Waals size of the molecule, a voxel sparsity (see Ref. (A1)), allowing only a finite non-zero number of voxels (800 voxels, i.e., 26% of the support), and the known symmetry of the respective gas phase molecular orbital (i.e., antisymmetry along the x, y and z axes for the HOMO, and antisymmetry along the x and z axes but symmetry along the y axis for the LUMO). Together, the prior information and measurement data yield constraints that define the feasibility space, where the likelihood of an orbital guess is given by the combined discrepancy with all constraints. This is quantified by the reconstruction gap, which is zero for a solution that is consistent with all data and prior knowledge. Starting with a random guess, the CP algorithm seeks to find a solution that minimizes this discrepancy by iteratively enforcing the respective constraints. In this way, CP converges to fixed points (local minima) of the feasibility landscape (A2). However, due to measurement noise and inconsistency between the different constraints, a solution that complies with all given constraints generally does not exist, and additionally, many local minima exist (A3). Therefore, as discussed in Section 5, we run many independently initialized reconstruction trials and assess their quality based on the reconstruction gap.

Comment 1.7

p.8 l.201ff For comparison with their POT results the author use DFT calculated orbitals of planar gas-phase PTCDA. A planar geometry is also assumed as a symmetry constraint in the CP algorithm. However, DFT of PTCDA/Ag(110) as well as experiments with x-ray standing waves suggest that the adsorbed PTCDA molecules adopt a bent geometry with the carboxylic oxygen atoms approx. 0.3 angstrom closer to the metal substrate than the perylene carbon backbone (e.g. Mercurio et al. PRB 87 045421 (A4)). In their earlier work [(A3)] the authors demonstrated that their method is in principle capable of reconstructing the wavefunction of a bent molecule. It would be interesting to see how the reconstructed orbitals would change if the symmetry constraint perpendicular to the substrate is released. Have the authors performed such calculations? A bent geometry could possibly also explain the slightly different shape of the reconstructed orbitals at the outer lobes compared with gas-phase DFT.

Answer 1.7: We agree with the referee that it would be highly interesting to employ our method to investigate the adsorption geometry and, in particular, deviations compared to the gas phase molecule. Unfortunately, the expected changes, where the oxygen atoms are ≈ 0.3 Å closer to the metal substrate than the perylene carbon backbone, are substantially smaller than the bending of the PTCDA molecule on MgO surface (around 1 Å), which we used for benchmarking in Ref. (A3). In the present case, the subtle bending of the molecule lies below our Fourier-related spatial resolution of 0.75 Å. We therefore expect that any effects of bending in the HOMO and LUMO momentum fingerprints are hard to observe. This is also supported by the data: we observe no photon-energy dependence in the position of the main lobes.

Nevertheless, to investigate the z-symmetry of the HOMO and LUMO orbitals, we have also done reconstructions without z-symmetry constraint. Such reconstructions are challenging because the increased parameter space leads to many new local minima. This necessitates a large number of random initializations (> 1000) to find the most likely orbital (i.e., the lowest gap), and even more reconstructions are needed to ensure reliable statistics. While 1000 of these reconstructions were inconclusive for the LUMO, the lowest-gap reconstruction that we found for the HOMO is not only consistent with the expected orbital shape, but also shows minimal deformations that might be consistent with a bonding-induced shift of the oxygen atoms. For the referees, this reconstruction is shown in Fig. A1. However, we emphasize that a significant improvement in algorithm efficiency is necessary before we could unambiguously claim to have observed the (non-)symmetric nature of the orbital.

Changes to the manuscript 1.7:

- Since the reconstructions without enforced z-symmetry do not allow stronger conclusions than observing that the curvature of the molecule is weak compared to our resolution, we have added the following note to the article (Section 4):

  > In this context, we note that a slight bending of the adsorbed PTCDA molecule has been reported, with the oxygen atoms positioned ≈ 0.3 Å closer to the substrate than the carbon backbone (A4). However, this distortion falls below the current spatial resolution of 0.75 Å, indicating that distortion-induced changes to the photoemission orbital tomography data will be very hard to observe. Since we detect no significant photon-energy dependence in the main lobe positions, and reconstructions without enforced z-symmetry constraints are consistent with a planar molecular geometry, we conclude that any influence of this bending on the HOMO and LUMO lies below our detection limit.

- We also added the following to section 5:

  > This also holds for the symmetry constraint, where we have observed that releasing z-symmetry leads to a large number of spurious local minima that so far prevented a definitive analysis of the potential small bending of the PTCDA molecule (A4).

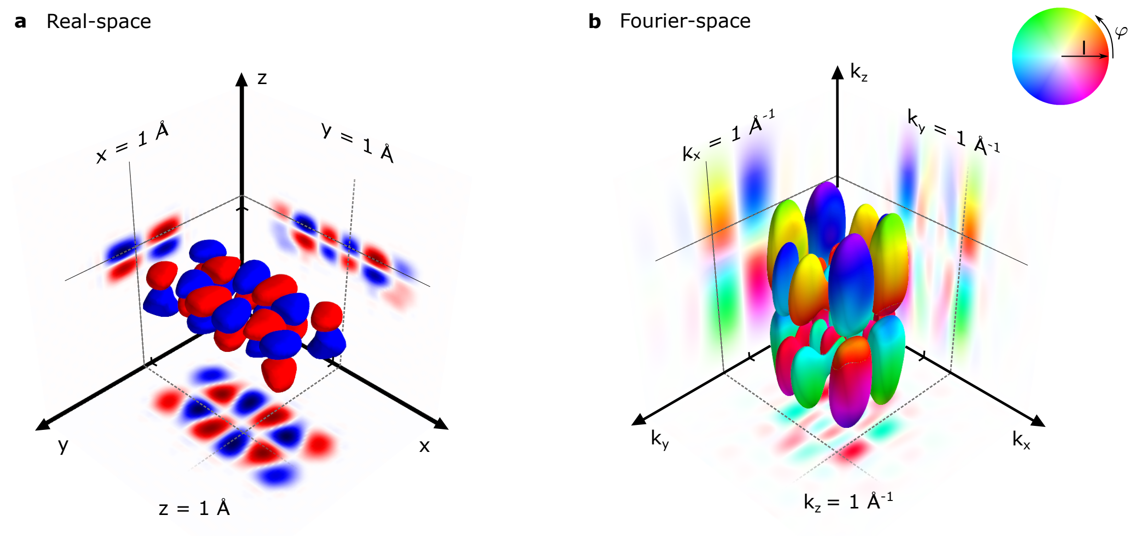

Figure A1: Tentative reconstruction result where z symmetry was *not* enforced. a) Isosurface representation of the best HOMO reconstruction out of a set of 1000 random initializations, together with slices at fixed x, y and z. Notably, the outermost lobes which are localized on the oxygen atoms show a downward distortion than could be interpreted to follow from bending of the PTCDA molecule. This is most visible in the fixed-y cut. b) Corresponding momentum space isosurface and cuts, where phase is encoded as color and amplitude by brightness. Due to the lack of symmetry, the phase profile is more complicated with values other than 0 or π also appearing.

Response to Reviewer 2

The ability to perform time resolved studies of molecular orbitals would be of significance to understanding the physics and chemistry of surface processes. The combination presented here of ultrafast momentum microscopy with an algorithm to reconstruct 3D orbitals, that the authors have developed, puts this within reach. Before publication the authors should consider the following.

Answer 2.0: We thank the reviewer for their positive review and their helpful comments below.

Comment 2.1

The summary of the constraints made during the orbital reconstruction on page 8 could be misconstrued to suggest that they have forced/assumed the result – this should be clarified.

P8..“In addition to the experimental data, the algorithm incorporates several constraints on the reconstructed orbital. Namely, we employed the known symmetry in the x, y and z directions, a loose support of $12 \times 18 \times 6$Å (i.e., roughly matching with the van der Waals size of the molecule) and voxel sparsity of the molecular orbital (800 voxels, i.e., 26% of the support).”

I do not believe the authors assumed the known symmetry of the orbital.

Answer 2.1: We understand the concerns of the referee regarding the use of prior knowledge about the molecular orbital for the reconstruction procedure. However, we would like to emphasize that although additional prior knowledge is required to compensate for the missing phase information, we do not assume complete knowledge of the molecular orbital, which would render the reconstruction obsolete. While we assume the maximal size of the molecular orbital, which we estimate by the van-der-Waals size of the molecule, and the symmetry, which can be determined by group theory (A5), we do not impose any detailed information on the orbital shape, such as

for example the number, sign or shape of the lobes. Therefore, we are convinced that our approach is valid to reconstruct the molecular orbital and is still independent of more involved theoretical calculations, such as DFT. Furthermore, we note that the assumed prior knowledge is commonly used in the POT community (see Refs. (A6; A7; A8; A1)).

As a side note, we also discuss the applicability of z symmetry in Comments 1.7 and 3.1.

Changes to the manuscript 2.1: In response to this question and also Comment 1.6, we now provide a more detailed description of the CP algorithm in the Method section 7.4. See our answer to Comment 1.6 for the full added text.

Comment 2.2

Due to the single incident geometry of the light an asymmetry in the photoemission distribution results (unlike the original static POT experiments where the photoemission was always recorded in the plane of incidence (and polarization plane) by rotating the sample). The experimental momentum distribution could either be artificially (but reasonably) symmetrised or an A.k factor could be applied which would lead to a symmetric distribution. What have the authors actually done?

Answer 2.2: The reviewer is, of course, correct that the left-right asymmetry in the experimental data follows from the $\vec{A} \cdot \vec{k}$ factor given by the single incidence angle. We realize that we did not clearly state how we deal with this asymmetry, and have now made the changes detailed below to address this. Specifically, we indeed use symmetrization and division by the remaining symmetrized $\vec{A} \cdot \vec{k}$ to get an optimal approximation of $|\Psi(\vec{k})|$ with minimal noise. In Figure 2, however, we prefer to show the unsymmetrized momentum maps to highlight the excellent data quality.

Changes to the manuscript 2.2: Section 3, added

> The observed left-right asymmetry in both experiment and theory stems from the polarization factor $|\vec{A} \cdot \vec{k}|^2$ in equation (1), with the light impinging from the left. To retrieve the momentum space amplitude $|\mathscr{F}(\psi)(\vec{k})|$ required for reconstruction, the data are first symmetrized and subsequently divided by the symmetrized polarization factor.

Comment 2.3

From the raw data it seems clear that the incident plane was not in a high symmetry direction- the experimental geometry should be given in the methods section.

Answer 2.3: We thank the reviewer for pointing out this missing information. We have added it in the Method section.

Changes to the manuscript 2.3: Methods, added "We used p-polarized light with a fixed incidence angle of 68° to the surface normal."

Comment 2.4

For both the HOMO and LUMO two equally probable orbitals are obtained that have different electron density distributions – the authors believe they can identify the correct result:

P10..." Here, we focus on those reconstructions where the momentum-space phase flips between adjacent lobes, since we estimate that the faulty reconstructions result from residual background noise that obscures the true zero amplitudes between these lobes. Incidentally, this choice is also in line with gas-phase DFT calculations.

Taking this into consideration, we emphasize that by analyzing the gap and the reconstructed momentum distribution, 3D orbital imaging can be performed without the need to perform additional DFT calculations".

Are they saying that they choose a result where the sign of the wavefunction changes between emission lobes. This works for the HOMO (in that it yields a result in agreement with the gas DFT orbital) but as I see it (fig S8 and S6) it yields the wrong result for the LUMO as the k-space gas phase DFT orbital does not have a phase change between the lobes.

Answer 2.4: The referee correctly points out that, in the original submission, the identification of the most likely orbital reconstruction relied partly on manual inspection, especially for the LUMO. In retrospect, this was a consequence of the imperfect intensity calibration used at that stage. As discussed in the General Remark, the previous calibration introduced systematic distortions in the relative intensities at different photon energies. While these distortions did not prevent the reconstruction of the correct qualitative orbital shape (as we know from our benchmarking in Ref. 27), they did modify the feasibility landscape of the reconstruction problem and thus affected the absolute values of the reconstruction gap. In that situation, the gap could no longer be used as a fully reliable metric to distinguish between competing reconstruction clusters, and we therefore had to rely more strongly on visual inspection and physical reasoning. We emphasize that such inspection of the measured momentum maps remains an important consistency check; however, in the original submission it played a disproportionately large role because the gap metric was compromised by the imperfect calibration.

With the improved intensity calibration implemented in the revised manuscript, this limitation is largely removed. As can be seen from the updated Figures 4, S5, and S7, the reconstruction clusters that agree with the DFT reference orbitals have shifted towards lower gap values and are now clearly favored. In particular, with 10 photon energies, a single reconstruction trial is now more likely to yield the correct orbital. Although the numerical differences in gap between clusters are still modest, they are consistent across all photon energies and with the measured momentum fingerprints. In the revised analysis, visual inspection of the momentum maps therefore serves mainly as a qualitative cross-check of the gap-based selection, rather than as the primary selection criterion. Taken together, this provides a more objective and internally consistent basis for identifying the most likely orbital without relying on DFT as an external constraint.

Changes to the manuscript 2.4:

- Figures 4, S5, and S7 have been updated with the new reconstructions based on the improved calibration. Moreover, we now indicate the average gap of the individual reconstruction clusters.
- We have added the following text to the caption of Figure 4:

  ... the cluster size, and $(10^3\times)$ the average gap $(\bar{g})$ ...

- We have adapted Section 5 with the following text:

  In momentum space, this mainly corresponds to a phase flip of the weaker amplitude peaks. A detailed comparison of these reconstructions is given in the Supplementary Information (Section 8.5 and Supplementary Fig. S8). From this analysis, we find that the lower-gap reconstructions match better to the measured data, and we therefore identified these reconstructions as the most likely true orbital. Incidentally, this choice is also in line with gas-phase DFT calculations. Thus, we emphasize that by analysing the gap and the reconstructed momentum distribution, 3D orbital imaging can be performed without the need to perform additional DFT calculations.

- We have reworked Section 8.5 of the Supplementary Information:

  For both the HOMO and the LUMO, a strong difference in the phase profile is accompanied by subtle changes in the amplitude profile, as highlighted by the arrow.
  For the HOMO, the first reconstruction cluster (with gap $= 1.41$) shows a phase flip between the two lobes, as well as a slight slanting of the lobe at $(k_x, k_y) \approx (0.5, 1.5)$ Å^{-1}. On the other hand, the second cluster (gap $= 1.42$) shows no zero crossing in the amplitude between these lobes. Comparison with Fig. 2 thus suggests a better match between the reconstruction and the

measurement data for the first cluster. For the LUMO, the first cluster (gap = 1.42) shows a weak, opposite-phase lobe that inserts between the stronger photoemission features, and also two elongations of the main (upper) lobe curving towards the center are seen. For the second cluster (gap = 1.42), only the zero-crossings and lobes show a significantly different shape. Comparison with Fig. 2 (particularly for 20.5 eV), supports the identification of first cluster as the most likely result. These results are also consistent with the expectation from DFT.

Response to Reviewer 3

In this manuscript, Bennecke et al. perform 3D photoemission orbital tomography (3D-POT) of ordered PTCDA films on Ag(110) using a laser-based XUV source and a time-of-flight momentum microscope. This POT technique uses several major approximations to interpret angle-resolved photoemission (ARPES) as "molecular orbital tomography." While the approximations give reasonable results for certain cases, for example the large polycyclic aromatic hydrocarbons used in this work, POT is not really a widely applied technique in either ground-state or excited-state (i.e. time-resolved) photoemission. The system studied here, PTCDA on Ag(110), has been extensively studied using both POT (e.g. refs 21 and 22 of the paper) and may other methods.

The main new things we see in the manuscript by Bennecke et al. is A) the use of a femtosecond XUV source, as opposed to a synchrotron, and B) the experimental demonstration of amplitude and phase reconstruction from sparse data using the methods described in ref 27 of the paper. The momentum microscope and 500 kHz HHG source are near the current state-of-the-art in tr-ARPES technology, and the authors do successfully reproduce 3D-POT of the PTCDA/Ag(110) system. The technical level is very high. However, in our opinion, although the experiments have been done using a femtosecond XUV source, the authors have not really demonstrated the feasibility of time-resolved 3D-POT, A). First of all, there is no time-resolved data in the paper. Second, the demonstrated acquisition times indicate that an excited-state experiment will be very very difficult. The authors state that one ground-state 3D-POT can (barely) be done with 4 photon energies in 8 hours of data acquisition. In excited state measurements, the signal will inherently be much lower. For example, to study dynamics that have any chemical or device relevance for this system, it is likely that the fraction of molecules excited should be 1% or even less to ensure linear dependence on the observed signals on the pump fluence. Thus the signal levels will be at least 100 times smaller, indicating more than 80 hours of acquisition to achieve the same signal levels for just one pump-probe delay. However, it is likely worse than this due to the background being significant, such that it may be that excited-state signal is even below the background. Furthermore, it seems from the results that using only 4 photon energies is likely insufficient to have a faithful reconstruction without additional constraints, inputs, or control experiments (e.g. more photon energies). Overall, we believe that the data presented actually shows that chemically relevant time-resolved 3D-POT using this setup is not feasible, at least with the currently demonstrated signal and background levels. Thus, if this is actually the first realization of B) it may be better to emphasize this more in the paper than A).

Overall, we find this paper a valuable contribution to the field in terms of its systematic approach and clear statements regarding integration times, faithfulness of reconstruction, etc. It is helpful in terms of understanding the signal levels and limitations of the technique. This is difficult experiment, and solid work in an overall difficult and competitive field. We would like to see this work published.

Some more specific comments are below:

Answer 3.0: We thank the reviewer for positive recommendation and constructive feedback.

We fully agree that a time-resolved 3D orbital imaging experiment at chemically relevant excitation densities is experimentally very demanding. Nevertheless, we do not share the conclusion that such experiments are infeasible. Beyond the data presented in the manuscript, our assessment is based on two considerations.

First, the background conditions in time-resolved measurements can differ favorably from those in static measurements. In the static case, the background is dominated by strong occupied substrate bands, whereas in pump–probe experiments the relevant signal can often be separated from background by its distinct temporal evolution. In previous work from our group on a PTCDA/WSe_2 heterostructure (A9), for example, we have demonstrated that transient molecular excitations can be extracted with sufficient signal-to-noise ratio from

overnight measurements at comparable repetition rates.

Second, the present dataset was acquired with a non-optimized, first-generation implementation of the setup. There is substantial room for improvement in EUV flux, focusing, and phase matching, as well as in the effective acquisition rate (e.g. via energy filtering in the momentum microscope and higher laser repetition rates up to 2 MHz). Taken together, these developments provide more than an order of magnitude potential gain in effective data throughput. We therefore view the present work as a necessary and encouraging step towards time-resolved 3D orbital imaging, rather than as a demonstration of its ultimate limitations.

Comment 3.1

The authors tried to reconstruct the 3D frontier orbital of PTCDA molecule from the PTCDA grown on Ag(110) surface with brick wall superstructure. They compare with gas-phase PTCDA calculations However, it has been shown that the strong interaction between PTCDA and Ag(110) surface will lead to the formation of chemical bond, which will deviate the LUMO of the adsorbed molecule from free molecule [Physical Review Letters, 2010, 104(23): 233004]. The chemical contribution also bends the molecular plane to favor the interaction of oxygen atoms with the Ag surface atoms underneath [PRB 2013, 87(4): 045421]. Therefore, the interfacial hybridization and molecular bending will complicate the reconstruction from sparse 3D POT data, and the influence of them should be addressed.

Answer 3.1: The referee is right, the PTCDA molecule adsorbed on the Ag(110) surface is prone to a slight bending. Particularly, as also pointed out by reviewer 1 (see comment 1.7), the oxygen atoms are expected to be approx. 0.3 Å closer to the metal substrate than the perylene carbon backbone (A4). In our current reconstruction approach, we neglect this subtle bending of the molecule by enforcing a symmetry constraint along the z-axis. In general, we agree that these adsorption-induced deviations from the DFT gas-phase molecule are highly interesting and well-suited to study using our approach. However, in this specific case, unfortunately, the bending of the molecule lies below our spatial resolution of approx. 0.75 Å, meaning that any related effects in the momentum map will be quite subtle. As detailed in the answer to Comment 1.7 and shown in Fig. A1, we have performed first reconstruction trials where no z symmetry was enforced. These reconstructions require further development of the algorithm to yield definitive data, but first results show that the orbital could indeed show asymmetry due to the displacement of the oxygen atoms. Due to the tentative nature of these results, we chose to include them only in the response letter.

Changes to the manuscript 3.1:

- Since the reconstructions without enforced z-symmetry do not allow stronger conclusions than observing that the curvature of the molecule is weak compared to our resolution, we have added the following note to Section 4:

> In this context, we note that a slight bending of the adsorbed PTCDA molecule has been reported, with the oxygen atoms positioned ≈ 0.3 Å closer to the substrate than the carbon backbone (A4). However, this distortion falls below the spatial resolution of 0.75 Å, indicating that distortion-induced changes to the photoemission orbital tomography data will be rather weak. Since we observe no significant photon-energy dependence in the main lobe positions, and reconstructions without enforced z-symmetry constraints are consistent with a planar molecular geometry, we conclude that any influence of this bending on the HOMO and LUMO lies below our detection limit.

Comment 3.2

Related to point 1, it is unclear what is going on in the z direction in figure 3. It seems that in figure 3e, z=0 may

correspond to the plane of the atoms (node in the pi orbitals). However, in figure 3c there is no axis labeled z, and the one that is likely z seems not centered on the nodal plane. Furthermore, why is figure 3e not continued to negative z? In reality we would not expect the orbital to be anti-symmetric around z=0 due to the strong impact of the substrate, as discussed in the references of point 1.

Answer 3.2: First, thanks for noticing the error in Fig. 3c: indeed the vertical axis should be labeled 'z'. We also note that the z axis is indeed shifted exactly so that the nodal (atomic) plane coincides with $z = 0$. This is so because of the symmetry constraint employed in the algorithm. (As a side note, in phase retrieval problems such as this, lateral translation of the object does not affect the measured momentum-space intensities, and so we are not sensitive to such shifts. The symmetry constraint merely fixes our point of reference to the nodal plane.)

Figure 3e is not continued to negative z because the reconstructed orbital is antisymmetric, as imposed by the reconstruction constraints. The $z < 0$ part, therefore, does not provide additional information. We agree that the impact of the substrate on the molecular orbitals, particularly in the presence of strong hybridization, is highly interesting and well-suited to study using our approach (see also our answers to Comments 1.7 and 3.1). However, in this specific case, we find that such substrate-induced changes are so weak that we cannot conclusively analyze them.

Changes to the manuscript 3.2:

- We have updated Fig. 3 to include the correct z-axis label. We have also updated Supplementary Figure 6, where the same error occurred.
- We have added ticks on each axis to indicate the zero position.

Comment 3.3

We do not find any quantitative comparison between the reconstructed k-space data (e.g. fig. 3b) and the measured data (e.g. fig 3a). It is thus difficult to determine how good the reconstruction is.

Answer 3.3: We appreciate the suggestion of the referee and have added an additional Supplementary Figure S9. Here we show a comparison of the experimentally measured k_z dependence and the reconstructed z dependence with the predictions from DFT.

Changes to the manuscript 3.3:

- Added Supplementary Figure S9.
- A section was added to the Supplementary Information:

  > In Supplementary Fig. S9, the k_z dependence is compared between the measured data, the reconstructed orbital, and the DFT prediction. These profiles were extracted in a small region centered on one of the main photoemission features of each orbital (see box in Supplementary Fig. S9a and d). Concerning the measured data, it is important to note that photoemission momentum maps were first embedded in the full 3D momentum space before a line-out was extracted. In this way, the strong curvature of the constant-energy hemisphere could be accounted for, and multiple k_z values could be extracted from a single momentum map at fixed photon energy.

- In Section 4, we added a reference to the new Supplementary Figure: "Additionally, Supplementary Figure S9 provides a quantitative comparison of measured and reconstructed k_z profiles with DFT calculations, demonstrating excellent agreement across all photon energies."

Comment 3.4

After processing and reconstruction(s), data is compared to Kohn-Sham DFT orbitals. However, strictly speaking these are not rigorously defined. Better is to use the Dyson orbital formalism, which are uniquely defined, as discussed by Krylov [J. Chem. Phys. 153, 080901 (2020)]. At a minimum, the paper should have some discussion of this subtlety, and how the presented orbitals are related to Dyson orbitals. Maybe in Koopman's approximation they are the same in this case using an uncorrelated electronic structure method, but it is not immediately clear.

Answer 3.4: The referee is correct; A more sophisticated description of the photoemission process from organic molecules is based on the use of Dyson orbitals as discussed by Refs. (A10; A11). However, as concluded in Ref. (A10) for PTCDA, the *"Dyson orbital approach leads to the same result as the molecular orbital approach and both are in agreement with the experimental data"*, which justifies our approach.

Changes to the manuscript 3.4: In section 4, we now explicitly discuss this: "(Note that a more accurate comparison employs Dyson orbitals, but these have been shown to match closely to KS orbitals for the present PTCDA system (A10; A11).)"

Comment 3.5

It is not clear what the XUV polarization direction is for the various presented data. It is also not clear how this accounted for in the theory. This could help explain asymmetries in the momentum maps, both in experiment and theory.

Answer 3.5: We thank the reviewer for pointing this out. For all photon energies, we use p-polarized light. In Fig. 2, we show the momentum maps as measured to highlight the excellent data quality, but for the reconstruction, we symmetrize the momentum maps and then correct for the remaining $|\vec{A}\cdot\vec{k}|$ factor.

Changes to the manuscript 3.5: As detailed in Comments 1.2, 2.2, we have included this information now in the manuscript:

- Section 3, added

  The observed left-right asymmetry in both experiment and theory stems from the polarization factor $|\vec{A}\cdot\vec{k}|^2$ in equation (1), with the light impinging from the left. To retrieve the momentum space amplitude $|\mathscr{F}(\psi)(\vec{k})|$ required for reconstruction, the data are first symmetrized and subsequently divided by the symmetrized polarization factor.

- Methods, added "We used p-polarized light with a fixed incidence angle of 68° to the surface normal."

Comment 3.6

For the background subtraction, the author has considered the Gaussian distribution of the orbital, the Fermi surface of Ag and some linear background. However, it seems there is still significant background in figures 2d and 2e. This problem has been dealt with previously [The European Physical Journal B, 2019, 92: 1-8] ((A12)), and has been shown to strongly influence reconstructions.

Answer 3.6: We are aware that the performed background subtraction is not perfect and still contains remaining contributions from the silver bands, as pointed out by the referee. We agree that this procedure could potentially be improved by employing lineshape separation (A12) or using reference data of the clean silver substrate (A8). As another approach, data points where substrate and molecular orbital signals cannot be disentangled well enough could be left unknown in the reconstruction algorithm, enabling the algorithm to recover these unknown data when enough other data points are present. However, we emphasize that the presented reconstruction algorithm yields a reliable reconstruction of the three-dimensional molecular orbital even when using only 4 different photon energies,

despite the remaining background. This holds even for the LUMO, where increased background features lead to a somewhat less valuable gap and reduced reconstruction accuracy for the weaker photoemission signatures, c.f. Supplementary Figure S9. Nonetheless, the successful reconstruction of the three-dimensional orbital even under these conditions demonstrates the robustness of the approach.

Changes to the manuscript 3.6:

- We added a line to Methods Section 7.3: "At some photon energies, a residual signal stemming from the Ag(110) substrate can also be found, which could potentially have an effect on the reconstructions (A12). However, in our case, we we find that this background does not noticeably affect the reconstruction quality."
- We slightly rephrased our description of the LUMO reconstruction in the main text: "We note that we observe a similarly good reconstruction of the LUMO (see Supplementary Fig. S6)"
- In the discussion of the LUMO reconstruction in the Supplementary Information, we now write: "We find excellent agreement between the in-plane (x, y) and out-of-plane (z) distribution, with minor differences that we attribute to increased background contributions in the momentum maps (A12)."

Comment 3.7

In Figure 3e about the z profile, does the yellow circle represent the measured data? If not, could the authors add the measured data after considering the polarization factor, as has been done in previous work [Nature communications, 2015, 6(1): 8287] ((A7))? Also, the reconstructed data displays a large deviation from the DFT data at about 2 angstroms, could the authors address the reason?

Answer 3.7: [See also the answer to Comments 1.4 and 1.5] The circles in Fig. 3e represent the data points retrieved from the orbital reconstruction at its native resolution. The solid lines are achieved by interpolation of these points. We cannot compare this real-space z-profile to the measured data since these are in different domains.

With regards to the large deviation previously observed around $z = 2$ Å, we emphasize that this deviation has disappeared in the revised data due to the improved intensity calibration carried out as part of this revision. (See also the General Remark above.)

Changes to the manuscript 3.7:

- To clarify the z profile plots, we have added the following information to the captions of Figure 3 and Supplementary Figure 6: "(yellow circles; the yellow line shows a Fourier interpolation.)" As detailed in the general remark, these figures have also been updated with new z-profiles.

Comment 3.8

(8a) Even with measurements at 10 photon energies, it seems that a spurious phase flip still exists in reconstructed data, so how can one distinguish the best reconstruction between the two phase flip results? If better background subtraction or more measured data is used, could this be avoided?

Answer 3.8: We agree with the reviewer that selection of the best (true) phase profile from these reconstructions is challenging. There are several factors that might contribute to this challenge: 1) a residual background signal can obscure regions in the momentum map where the Fourier amplitude drops to zero, thereby potentially obscuring sign flips. A similar effect could be caused by blurring or limited resolution of the momentum map. However, another challenge could be of pure, mathematical nature: for the given amplitude and phase retrieval problem and for the present degree of prior knowledge (where we do not know the exact support), it is not guaranteed that a unique solution exists - even when no experimental noise would be present.

Nevertheless, after we improved our intensity calibration, the situation improved significantly. As identified

in Ref. (A3), an error in the calibration can reduce the quality of the gap as a predictor for the most accurate reconstruction. This is directly reflected in our data: due to the improved calibration, the clusters and gaps in Figures 4, S5 and S7 changed slightly, meaning that the expected orbital (based on DFT) now always shows the lowest gap. Since the difference in gap is often very small, we combine this analysis with a careful inspection of the momentum space distribution (see updated SI Section 8.5 below).

Changes to the manuscript 3.8:

- Figures 4, S5, S7, and S8 have been updated following the new calibration data.
- We have adapted section 5 with the following text:

  In momentum space, this mainly corresponds to a phase flip of the weaker amplitude peaks. A detailed comparison of these reconstructions is given in the Supplementary Information (Section 8.5 and Supplementary Fig. S8). From this analysis, we find that the lower-gap reconstructions match better to the measured data, and we therefore identified these reconstructions as the most likely true orbital. Incidentally, this choice is also in line with gas-phase DFT calculations. Thus, we emphasize that by analysing the gap and the reconstructed momentum distribution, 3D orbital imaging can be performed without the need to perform additional DFT calculations.

- We also reworked Section 8.5 of the Supplementary Information:

  For both the HOMO and the LUMO, a strong difference in the phase profile is accompanied by subtle changes in the amplitude profile, as highlighted by the arrow.
  For the HOMO, the first reconstruction cluster (with gap $= 1.41$) shows a phase flip between the two lobes, as well as a slight slanting of the lobe at $(k_x, k_y) \approx (0.5, 1.5)$ Å^{-1}. On the other hand, the second cluster (gap $= 1.42$) shows no zero crossing in the amplitude between these lobes. Comparison with Fig. 2 thus suggests a better match between the reconstruction and the measurement data for the first cluster. For the LUMO, the first cluster (gap $= 1.42$) shows a weak, opposite-phase lobe that inserts between the stronger photoemission features, and also two elongations of the main (upper) lobe curving towards the center are seen. For the second cluster (gap $= 1.42$), only the zero-crossings and lobes show a significantly different shape. Comparison with Fig. 2 (particularly for 20.5 eV), supports the identification of first cluster as the most likely result. These results are also consistent with the expectation from DFT.

Comment 3.9

(8b) In terms of item B) mentioned above being potentially the more impactful aspect of the paper, it would be helpful for the authors to comment what level of data quality would be necessary to believe orbital reconstructions without any theoretical orbitals to compare to, if this is actually achievable.

Answer 3.9: We appreciate the reviewer's interest in 3D orbital imaging from sparse POT data, and we completely agree that this can bring powerful benefits to many experiments, also those not working with femtosecond dynamics. However, we note that it is challenging, if not impossible, to provide a general statement on the required data quality. In POT, the quality requirement depends on the application: in some cases, determination of the peak positions could suffice (A13), whether other experiments might require the measurement of more subtle changes to characterize distortion of a molecule (A14). Likewise in orbital imaging, the data quality must fit to the strength of the observed effect. Moreover, the shape of the orbital plays a role, e.g., since the spurious phase flip encountered for PTCDA orbitals was not observed for pentacene orbitals yet.

Despite these considerations, we emphasize that our current reconstructed orbitals are already at a level that the result can be directly used for the interpretation. For example, we can directly conclude from our reconstructions (which include reconstruction trials where no z-symmetry was enforced) that no strong curvature is present in the molecule.

Response to Reviewer 4

I co-reviewed this manuscript with one of the reviewers who provided the listed reports. This is part of the Nature Communications initiative to facilitate training in peer review and to provide appropriate recognition for Early Career Researchers who co-review manuscripts.

Answer 4.0: We thank the reviewer for their positive and constructive feedback.

Bibliography

References

[A1] Jansen, M. et al. Efficient orbital imaging based on ultrafast momentum microscopy and sparsity-driven phase retrieval. New J. Phys. (2020).

[A2] Luke, D. R., Thao, N. H. & Tam, M. K. Quantitative convergence analysis of iterated expansive, set-valued mappings. Mathematics of Operations Research **43**, 1143–1176 (2018).

[A3] Dinh, T. L., Jansen, G. S. M., Luke, D. R., Bennecke, W. & Mathias, S. A minimalist approach to 3D photoemission orbital tomography: algorithms and data requirements. New J. Phys. **26**, 043024 (2024). URL `https://dx.doi.org/10.1088/1367-2630/ad3e22`.

[A4] Mercurio, G. et al. Adsorption height determination of nonequivalent c and o species of ptcda on ag(110) using x-ray standing waves. Phys. Rev. B **87**, 045421 (2013). URL `https://link.aps.org/doi/10.1103/PhysRevB.87.045421`.

[A5] Mulliken, R. S. Electronic structures of polyatomic molecules and valence. iv. electronic states, quantum theory of the double bond. Phys. Rev. **43**, 279–302 (1933). URL `https://link.aps.org/doi/10.1103/PhysRev.43.279`.

[A6] Puschnig, P. et al. Reconstruction of Molecular Orbital Densities from Photoemission Data. Science **326**, 702–706 (2009). URL `https://science.sciencemag.org/content/326/5953/702`.

[A7] Lüftner, D. et al. Imaging the wave functions of adsorbed molecules. PNAS **111**, 605–610 (2014). URL `https://www.pnas.org/content/111/2/605`.

[A8] Kliuiev, P. et al. Algorithms and image formation in orbital tomography. Phys. Rev. B **98**, 085426 (2018).

[A9] Bennecke, W. et al. Hybrid Frenkel–Wannier excitons facilitate ultrafast energy transfer at a 2D–organic interface (2025). URL `https://www.nature.com/articles/s41567-025-03075-5`.

[A10] Dauth, M. et al. Angle resolved photoemission from organic semiconductors: Orbital imaging beyond the molecular orbital interpretation. New Journal of Physics **16**, 103005 (2014).

[A11] Krylov, A. I. From orbitals to observables and back. J. Chem. Phys. **153**, 080901 (2020). URL `https://pubs.aip.org/aip/jcp/article/1061735`.

[A12] Graus, M. et al. Three-dimensional tomographic imaging of molecular orbitals by photoelectron momentum microscopy. Eur. Phys. J. B **92**, 80 (2019).

[A13] Haag, F. et al. Momentum and energy dissipation of hot electrons in a Pb/Ag(111) quantum well system. Phys. Rev. B **104**, 104308 (2021).

[A14] Hurdax, P. et al. Large Distortion of Fused Aromatics on Dielectric Interlayers Quantified by Photoemission Orbital Tomography. ACS Nano **16**, 17435–17443 (2022).

We thank all reviewers and the editor very much for the constructive feedback on our manuscript. In the following, we reply step by step to the comments of the reviewers. A direct response to the reviewers is printed in **blue**, changes to the manuscript are printed in **red**.

Response to Reviewer 1

By employing a refined photon energy-dependent intensity calibration, the authors present photoemission data of even higher quality, enabling an improved reconstruction of the molecular frontier orbitals. In particular, the reconstructed z-dependence shows better agreement with predictions from DFT, which further strengthens the manuscript's main conclusion – that orbital reconstruction from sparse photoemission data is feasible.

I also appreciate that the authors tried reconstructions without imposing a z-symmetry constraint. It is particularly interesting to observe that the lowest-gap reconstruction of the HOMO appears to capture the slightly bent molecular geometry of PTCDA/Ag(110).

The authors have addressed all of my previous comments satisfactorily in the revised manuscript. Therefore, I recommend publication in its present form.

Answer 1.0: We thank the reviewer very much for the positive assessment of our work.

Response to Reviewer 2

The authors have satisfactorily responded to the original report and in doing so have improved the manuscript – I can now recommend it for publication.

Answer 2.0: We thank the reviewer for their positive evaluation.

Response to Reviewer 3

The authors have done substantial work here to improve the paper. Overall, it is a very good contribution to the field. The various quantitative comparisons shown give the expert a good sense of the status of the state of the art. This is important work.

Our main concerns regard the effect on the non-expert. We still feel several places in the paper the quality and impact of the results is overstated. While, unfortunately, overstatement is the norm these days in scientific writing, we feel for this subject in particular, it is dangerous. Orbital imaging with photoemission (time-resolved or not) is still very difficult and limited. The paper of Itatani (ref. 19 of this work, also in Nature...), is a prime example of overstatement in the orbital imaging business, and its negative effects are still felt today. "High-harmonic spectroscopy" never amounted to very much, but many people wasted a lot of time on it, and honest practitioners continue to fight the perception that ultrafast (even attosecond) orbital tomography is already a routine thing.

Answer 3.0: We thank the reviewer for the thoughtful and constructive follow-up report and for acknowledging the substantial work that went into improving the manuscript. We are fully convinced of the impact of our results and that time-resolved 3D orbital imaging is in reach, while we share the reviewer's concern regarding the wording and the potential of misunderstanding. We thank the referee for bringing this up and are happy to revise accordingly.

Comment 3.1

The following changes should be made:

1) The feasibility of time-resolved 3D POT at chemically-relevant excitation densities is at this point a quantitative question the authors can address relatively easily. If the authors want to continue to claim this is "within reach", they should quantitatively estimate what the integration times and required XUV exposures need to be, given the "optimized" experiment with 2 MHz repetition rate, less background for excited states, etc. They can do this, for example, by running a simulation (e.g. Monte Carlo) with the appropriate signal (e.g. 1% of the current signal level), background, etc. including multiple pump/probe delays. A quantitative estimate is more valuable than the various 'within reach' statements. In any event, this would give statements currently in the paper the appropriate context. Example statements include:

Line 74: "In this article, we overcome all of these limitations and demonstrate how 3D-POT can be performed in the laboratory at measurement durations that even permit the application of ultrafast 3D orbital imaging."

Line 86: "Our results clearly confirm the potential of table-top 3D-POT for systematic studies of orbital hybridization and even for full, femtosecond time-resolved orbital tomography"

Line 233: "promising to study dynamical processes with femtosecond time resolution, too."

Answer 3.1: We thank the reviewer for these suggestions. We find that a reliable simulation is challenging due to the large number of parameters that must be accounted for. In particular, it is not only necessary to estimate the strength of the dynamic signal, but also that of the background. We provide some examples for clarity:

- Assume that the excited-state signal lies far above the Fermi level, with no other signals present. In Ref. (A1), we studied photoelectron orbital imaging in the background-free case, where we simulated momentum maps with low total numbers of photoelectrons. In this case, our simulations showed that about 10^4 photoelectrons enable 2D orbital imaging, and additional (unpublished) tests indicated that even 10^3 counts can be sufficient. For comparison, the orbital momentum maps in our present measurements contain $\approx 10^6$ counts accumulated over 2 hours. This indicates that even at rather low excitation rates $< 10^{-3}$, excited state data can be reliably measured within a few hours. Additionally, the measurement time can be further reduced by increasing the repetition rate of the laser system, enabling the mapping of states with even lower excitation rates or allowing for a higher number of time steps to be acquired.

- Commonly, additional excited states will be present. Here, the necessary measurement time depends on the specifics of the excitation as well as the corresponding matrix elements. In our recent publication ((A2)), we have shown that the extraction of a state-specific momentum fingerprint was possible at a single photon energy based on an 8-hour measurement run. Repeating such a measurement for 4 photon energies would correspond to a total measurement time of ≈ 32 hours, which is well within the range of typical campaigns with our current experimental setup.

- Alternatively, ultrafast structural dynamics might be excited where a large fraction or even all of the molecules participate. Such a collective excitation could be observed in the occupied states, e.g., as a renormalization of the HOMO. For the case that all molecules are transiently modified, our results demonstrate that an integration time of two hours and four photon energies per time step are sufficient for a complete 3D reconstruction of the molecular orbital. For ten time steps, this corresponds to a total accumulation time of 80 hours, which is comparable to other momentum microscopy studies (e.g., (A3), appr. 70 hours). The data quality presented here (see Fig. 2 of the main manuscript) even suggests that the integration time per photon energy and time step could be further reduced while still enabling full 3D reconstruction. Increasing the repetition rate of the laser system to 2 MHz would further reduce the required measurement time by a factor of four.

  These considerations make us confident that time-resolved 3D reconstruction of the transiently modified orbitals is feasible, even if only a fraction of the molecules participates in optically induced ultrafast dynamics. In this case, the transient signal must exceed the noise floor of the static background and have a sufficiently high count rate. To be more quantitative, for a fixed in-plane momentum $\vec{k}$, we can model the total signal as $S(t) = T(t) + B$, where T is the time-dependent (transient) signal and B the time-independent background. Given that $T(t<0) = 0$, we extract the transient signal using $T(t) = S(t) - S(t<0)$. The noise

level (assuming Poisson statistics) is $\sqrt{S(t)} \approx \sqrt{B}$, where we estimate that the background is much stronger than the transient signal. It follows that the signal-to-noise ratio (SNR) $= T(t)/\sqrt{B}$. We will assume that $\mathrm{SNR} \geq 1$ is needed on average to reliably detect the transient signal. At typical background levels of $B \approx 10^2$ counts per energy-momentum voxel (based on the presented data), the noise floor is $\sqrt{B} \approx 10$ counts. If we then assume that 50% of the static photoelectron signal is due to the molecular layer (i.e., 50 counts per energy-momentum voxel), fractional excitations of $\gtrapprox 20\%$ could be detected. Indeed, a recent study (Ref. (A4)) reported such a collective motion where around 80% of the molecules participate, supporting the validity of this assumption for realistic sample systems. Since the assumed static background levels are comparable to those in our existing measurements, a transient measurement could be performed by starting with a measurement of the background at $t < 0$, and then measuring sets of spectra at 4 photon energies for ≈ 8 hours per pump-probe delay. Of course, the details of this calculation will strongly depend on the type of renormalization, which could involve energy shifts and/or momentum shifts.

We emphasize that such estimated measurement periods are indeed experimentally realistic with our experimental setup, where extended runs are feasible and competition for beam time is absent. At the same time, we note that these are illustrative scenarios; the actual requirements will depend sensitively on the specific sample, excitation conditions, and observable of interest.

We trust that this quantitative context, together with the reworded claims (see below), fully resolves the referee's concern about potential overstating, and illustrates the genuine potential of the approach.

Changes to the manuscript 3.1: We have reworded the following lines in the manuscript:

- line 22, modified text: "This invention opens a route towards ultrafast three-dimensional (3D) POT, with many new prospects for the study of ultrafast light-matter interaction, femtochemistry and photo-induced phase transitions."
- line 75, modified text: "how 3D-POT can be performed in the laboratory at dramatically reduced measurement durations".
- line 86, modified text: "Our results illustrate the potential of table-top 3D-POT for systematic studies of orbital hybridization and suggest a viable route towards future femtosecond time-resolved orbital tomography experiments."
- line 233, modified text: "Thus, we present the first realization of a table-top 3D-POT experiment, which, due to the use of ultrashort pulses from HHG, may enable future studies of dynamical processes with femtosecond time resolution."
- line 309: We have added a quantitative estimation of integration times: "Taking the current exposure time of 2 hours per photon energy as an example, we can see that 4 photon energies $\times$ 10 pump-probe delay steps would yield a total measurement time of 80 hours that is comparable to other momentum microscopy studies (e.g., (A3), appr. 70 hours)."
- line 305, we replaced: "This obviously leads to a tremendous reduction of the total required data collection and thus measurement time." with "At the same time, this initial measurement provides a precise characterization of the background that can be harnessed in subsequent measurements. This leads to a tremendous reduction of the total required data collection and aids in the identification of weak excited-state signals, thereby supporting the recording of photon energy-dependent dynamics."
- line 319: "We supplemented this setup by tailored orbital reconstruction algorithms, which allow efficient 3D data extraction and may enable ultrafast time-resolved studies in the future."

Comment 3.2

2) Figure S9 is very good, addressing the quality of the reconstruction in momentum space. The figure A1 in the response is also good, addressing our concerns regarding the ability of the reconstruction to discern asymmetry in the z direction. The authors should add A1 to the supplemental material.

Answer 3.2: We are grateful that the reviewer finds Figure S9 and the additional response figure A1 helpful in assessing the reconstruction quality and the sensitivity to z-asymmetry. However, we stress that Figure A1 presents just a single reconstruction. From the current reconstruction results, it is impossible to conclude whether such a bent orbital yields a better fit to the data or not. As a consequence, we strongly prefer to keep Fig. A1 in the review replies, where it will be publicly accessible within a suitable context.

Comment 3.3

3) The ability to reconstruct orbitals without guidance from DFT seems still overstated. The authors should remove the statement "Thus, we emphasize that by analysing the gap and the reconstructed momentum distribution, 3D orbital imaging can be performed without the need to perform additional DFT calculations." (line 263).

Changes to the manuscript 3.3: We have modified the line to "Thus, we emphasize that by analysing the gap and the reconstructed momentum distribution, accurate 3D orbital images can be extracted from the data with only limited prior information."

Response to Reviewer 4

I co-reviewed this manuscript with one of the reviewers who provided the listed reports. This is part of the Nature Communications initiative to facilitate training in peer review and to provide appropriate recognition for Early Career Researchers who co-review manuscripts.

Answer 4.0: We thank the co-reviewer for their contribution to the evaluation of our manuscript.

Bibliography

References

[A1] Jansen, M. et al. Efficient orbital imaging based on ultrafast momentum microscopy and sparsity-driven phase retrieval. New J. Phys. (2020).

[A2] Bennecke, W. et al. Hybrid Frenkel–Wannier excitons facilitate ultrafast energy transfer at a 2D–organic interface (2025). URL `https://www.nature.com/articles/s41567-025-03075-5`.

[A3] Bennecke, W. et al. Disentangling the multiorbital contributions of excitons by photoemission exciton tomography. Nat Commun **15**, 1804 (2024). URL `https://www.nature.com/articles/s41467-024-45973-x`.

[A4] Baumgärtner, K. et al. Femtosecond concerted rotation of molecules on a 2D material interface. Nature Communications **17**, 2110 (2026). URL `https://www.nature.com/articles/s41467-026-69801-6`.
